# BRD4 Degradation Enhanced Glioma Sensitivity to Temozolomide by Regulating Notch1 via Glu‐Modified GSH‐Responsive Nanoparticles

**DOI:** 10.1002/advs.202409753

**Published:** 2024-11-15

**Authors:** Linbin Yi, Zhenyu Zhang, Wenjie Zhou, Yunchu Zhang, Yuzhu Hu, Anjie Guo, Yongzhong Cheng, Zhiyong Qian, Peizhi Zhou, Xiang Gao

**Affiliations:** ^1^ Department of Neurosurgery and Institute of Neurosurgery State Key Laboratory of Biotherapy and Cancer Center West China Hospital West China Medical School Sichuan University and Collaborative Innovation Center for Biotherapy Chengdu 610041 China; ^2^ Department of Plastic and Burn Surgery West China School of Medicine West China Hospital Sichuan University Chengdu 610041 China; ^3^ Department of Laboratory Medicine West China Second University Hospital Sichuan University Chengdu 610041 China; ^4^ Key Laboratory of Birth Defects and Related Diseases of Women and Children (Sichuan University) Ministry of Education Chengdu 610041 China

**Keywords:** ARV‐825, glioma chemotherapy, notch1, target delivery, temozolomide

## Abstract

Temozolomide (TMZ) serves as the principal chemotherapeutic agent for glioma; nonetheless, its therapeutic efficacy is compromised by the rapid emergence of drug resistance, the inadequate targeting of glioma cells, and significant systemic toxicity. ARV‐825 may play a role in modulating drug resistance by degrading the BRD4 protein, thereby exerting anti‐glioma effects. Therefore, to surmount TMZ resistance and achieve efficient and specific drug delivery, a dual‐targeted glutathione (GSH)‐responsive nanoparticle system (T+A@Glu‐NP) is designed and synthesized for the co‐delivery of ARV‐825 and TMZ. As anticipated, T+A@Glu‐NPs significantly enhanced penetration of the blood‐brain barrier (BBB), facilitated drug uptake by glioma cells, and exhibited efficient accumulation in brain tissue. Additionally, T+A@Glu‐NPs exhibited augmented efficacy against glioma both in vitro and in vivo through the induction of apoptosis, inhibition of proliferation, and cell cycle arrest. Furthermore, mechanistic exploration revealed that T+A@Glu‐NPs degraded the BRD4 protein, leading to the downregulation of Notch1 gene transcription and the inhibition of the Notch1 signaling pathway, thereby augmenting the therapeutic efficacy of glioma chemotherapy. Taken together, the findings suggest that T+A@Glu‐NPs represents a novel and promising therapeutic strategy for glioma chemotherapy.

## Graphical abstract

In this study, a Glu‐modified and GSH‐responsive nanoparticle is constructed to enhanced drug delivery and targeting capabilities. Sugar modification helps nanoparticles penetrate the BBB and target gliomas, eventually responsively releasing the drug in the high GSH environment within the glioma cells. Encapsulating TMZ and ARV‐825 within nanoparticles can enhance the chemotherapeutic efficacy of TMZ in gliomas.

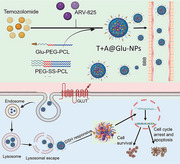

## 1 Introduction

The infiltrative growth pattern of gliomas frequently precludes complete surgical resection of the tumor, making chemotherapy as an indispensable component in the treatment regimen for gliomas.^[^ ^1^, ^2^ ^]^ Temozolomide (TMZ), as a clinical first‐line chemotherapeutic agent for gliomas, is capable of inducing DNA damages of glioma cells to further kill residual tumor tissue.^[^ ^3^, ^4^ ^]^ Nonetheless, the median survival for the majority of glioma patients remains less than two years, potentially due to the rapid development of drug resistance, inadequate targeting of glioma cells, and systemic toxicity.^[^ ^5^, ^6^, ^7^, ^8^, ^9^ ^]^ Thus, it is urgent to explore novel drug combinations and delivery strategies to overcome these obstacles.

The development of resistance to TMZ poses unprecedented challenges in the chemotherapy of gliomas. However, the mechanisms underlying TMZ resistance in gliomas remain insufficiently explored.^[^ ^10^, ^11^, ^12^ ^]^ Accumulating researches show that bromodomain‐containing protein 4 (BRD4) is upregulated in various solid tumors including head and neck, breast and brain tumors,^[^ ^13^, ^14^, ^15^ ^]^ in which BRD4 protein could regulate certain oncogenes expression to participate in tumorigenesis.^[^ ^16^, ^17^ ^]^ Furthermore, some studies have demonstrated that BRD4 protein would upregulate the O6‐methylguanine‐DNA methyltransferase (MGMT)/DNA repair pathway to repair the DNA damage caused by chemotherapy drugs.^[^ ^18^, ^19^, ^20^ ^]^ In addition, numerous bromodomain and extra‐terminal domain (BET) inhibitors have been employed in tumor chemotherapy, exhibiting some efficacy and conferring a survival benefit for glioma patients.^[^ ^21^, ^22^, ^23^ ^]^ All of the evidences showed that the BRD4 protein, as an epigenetic regulation factor, may be a potential and novel target to enhance the efficacy of TMZ in glioma treatment. However, most small‐molecule inhibitors of the BRD4 protein are more susceptible to drug resistance and have severe side effects due to their high dosage requirements, low selectivity, and transient effects. To overcome these challenges, the novel proteolysis‐targeting chimera (PROTAC) strategy has been developed, which offers greater selectivity and can induce efficient degradation with minimal quantities.^[^ ^24^, ^25^ ^]^ Notably, ARV‐825, a BRD4 PROTAC, has been shown to significantly inhibit glioma growth at nanomolar concentrations by inducing apoptosis and inhibiting cell proliferation, thereby further corroborating this notion.^[^ ^26^ ^]^ Consequently, we endeavored to incorporate ARV‐825 as an adjuvant in TMZ‐based glioma chemotherapy.

Although novel drug combinations may sensitize glioma chemotherapy, low blood‐brain barrier (BBB) penetration and poor glioma targeting can impair the efficacy of the drugs.^[^ ^27^, ^28^, ^29^ ^]^ To overcome these hindrances, large number of researches had focused on the development of advanced BBB crossing strategies, such as receptor‐mediated transcytosis, the use of neurotropic viruses, nanoparticles and exosomes.^[^ ^30^, ^31^, ^32^, ^33^ ^]^ Compared to other strategies, receptor‐mediated transcytosis is more sensitive and specific for drug delivery, as numerous receptors are highly expressed on the cerebrovascular endothelium and can facilitate drug delivery systems with corresponding ligand modifications to cross the BBB, such as transferrin receptor (TfR), insulin and insulin‐like growth factor receptor (IGFR), low density lipoprotein receptor (LDLR), and glucose transporter (GLUT).^[^ ^34^, ^35^, ^36^ ^]^ Among these, the GLUTs are highly expressed on both the cerebrovascular endothelium and the cell membrane surface of glioma cells, which can promote the penetration of glucose through the BBB and its subsequent uptake by glioma cells. Therefore, a number of Glu‐modified drug delivery nanoparticles have been verified to indeed facilitate blood‐brain barrier penetration and rapid uptake by gliomas, thereby enhancing the efficacy of drugs.^[^ ^37^, ^38^ ^]^

**Scheme 1:**
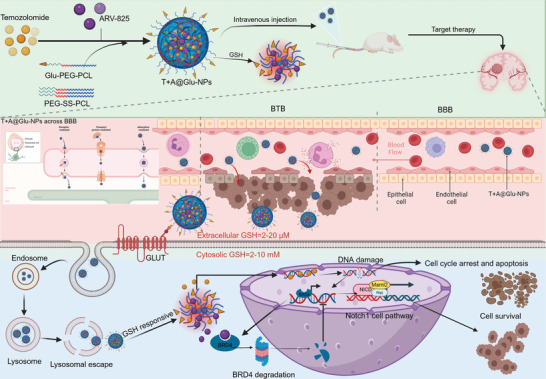
Schematic illustration of the brain‐targeting and therapeutic mechanism of T+A@Glu‐NPs against glioma. Glu‐PEG‐PCL, PEG‐SS‐PCL, TMZ, and ARV‐825 were used to prepare T+A@Glu‐NPs nanoparticles via a self‐assembly method. Administered intravenously, T+A@Glu‐NPs leverage the glucose transporters expressed by the cerebral microvascular endothelium to penetrate the blood‐brain barrier and reach the tumor site. They then bind to the transporters highly expressed on the surface of glioma and are efficiently internalized into the cells. After lysosomal escape and response to glutathione (GSH) reduction, T+A@Glu‐NPs disintegrate and rapidly release TMZ and ARV‐825. TMZ acts on DNA to exert its cytotoxic effect, while ARV‐825 induces the sustained degradation of BRD4 protein through the ubiquitin‐proteasome pathway, thereby downregulating Notch1 and its associated signaling pathways. This further enhances the apoptotic and cell cycle arrest effects induced by TMZ in glioma cells, achieving a more potent glioma‐killing effect.

Inspired by previous explorations, a Glu‐modified nanoparticle (T+A@Glu‐NPs) was designed and constructed for combined delivery of ARV‐825 and TMZ for the glioma treatment in this study (**Scheme** **1**). The T+A@Glu‐NPs consist of Glu‐modified nano micelles, Glu‐PEG‐PCL, and glutathione (GSH)‐responsive nano micelles, PEG‐SS‐PCL, which form the shell of the nanoparticles. ARV‐825 and TMZ were encapsulated in the hydrophobic core. Elaborate experiments were designed to characterize and validate the function of T+A@Glu‐NPs. It is revealed that glucose modification significantly augmented blood‐brain barrier penetration and facilitates drug uptake by glioma, and T+A@Glu‐NPs rapidly disintegrated to release the drugs in the high GSH environment within glioma, resulting in effective intracranial accumulation of the drug. In vitro anti‐glioma experiments were conducted to analyze the effects of the combined drugs on cell proliferation, cell cycle, cell apoptosis and other tumor biological behaviors. In addition, the therapeutic effects of the combined drug groups were evaluated in three murine models of glioma. Further exploration of the underlying mechanisms disclosed that BRD4 protein binds to the Notch1 promoter region, regulates its transcriptional expression and leads to TZM treatment sensitization. In conclusion, our studies demonstrated that the Glu‐modified and GSH‐responsive nanoparticles enhanced drug delivery and targeting capabilities. Therefore, the T+A@Glu‐NPs represents a novel and promising drug combination strategy, offering new insights into the clinical treatment of glioma.

## 2 Results

### 2.1 Preparation and Characterization of T+A@Glu‐NPs

**Figure 1:**
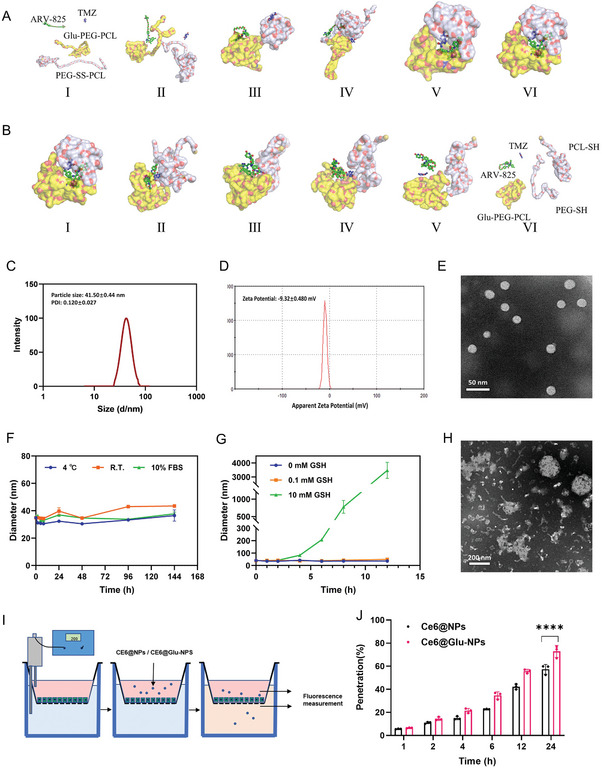
Characterizations of T+A@Glu‐NPs. Molecular dynamics simulation analysis A) the self‐assembly of TMZ, ARV‐825 and Glu‐PEG‐PCL / PEG‐SS‐PCL in the normal physiology environment (PH 7.4, GSH 0.1 mM). B) the GSH responsive and drug release process of T+A@Glu‐NPs in high GSH environment (GSH 10 mM). Conformations (I), (II), (III), (IV), (V), and (VI) represented the snapshots of the interaction between TMZ, ARV‐825 and Glu‐PEG‐PCL/PEG‐SS‐PCL at 0, 4, 8, 12, 16 and 20 ns, respectively. C) Particle size distribution of T+A@Glu‐NPs. D) Zeta potential of T+A@Glu‐NPs. E) TEM image of T+A@Glu‐NPs (scale bar = 50 nm). F) The stability experiment of T+A@Glu‐NPs. G) The glutathione reductive responsive release of TMZ and ARV‐825 from T+A@Glu‐NPs. H) TEM image of the reductive response morphological changes of T+A@Glu‐NPs. (I) Single‐layer BBB (Blood‐Brain Barrier) model penetration experiment of T+A@Glu‐NPs. (J) Statistical analysis of the BBB penetration rate. Data are presented as mean ± SD. No significant difference is marked with ns. *P < 0.05, **P < 0.01, ***P < 0.001 and ****P < 0.0001.

Glu‐modified nano micelles (Glu‐PEG‐PCL) and glutathione (GSH)‐responsive nano micelles (PEG‐SS‐PCL) were used to encapsulate ARV‐825 and TMZ, to increase the water solubility and stability of the drugs, and to enhance the targeting ability of this nanoparticle and its ability to penetrate the BBB. To further identify the interaction process of Glu‐PEG‐PCL, PEG‐SS‐PCL, ARV‐825, and TMZ, molecular dynamics simulation was performed to investigate their interactions in normal physiological environment (pH 7.4, GSH 0.1mM) and tumor‐mimicking high GSH environment (GSH 10mM). In normal physiological environments, ARV‐825 and TMZ presented constant change in position and conformations while interacting with Glu‐PEG‐PCL and PEG‐SS‐PCL within 20 ns, and gradual formation of tight interactions between drugs and nano micelles, ultimately forming stable drug‐loaded nanoparticles (**Figure** **1**A; Figure S1A, Supporting Information). And in tumor‐mimicking high GSH environment, after shedding PEG in response to the breakage of PEG‐SS‐PCL disulfide bond, the drug and nano micelles detached from each other to promote the release of the drug within 20 ns (Figure 1B; Figure S1B, Supporting Information). Then, we synthesized the PEG‐SS‐PCL and modified glucose to MAL‐PEG‐PCL to form targeted Glu‐PEG‐PCL (Figure S2A–C, Supporting Information), whose structure was confirmed via infrared spectroscopy analysis (Figure S3, Supporting Information). Subsequently, the drug loaded nanoparticles, termed as T+A@Glu‐NPs, was fabricated via self‐assembly of PEG‐SS‐PCL, Glu‐PEG‐PCL, ARV‐825 and TMZ. Detection of dynamic light scattering (DLS) showed that T+A@Glu‐NPs had a small particle size (41.50 ± 0.44 nm) and polydispersity index (0.120 ± 0.027), and zeta potential of ‐9.32±0.480 mV (Figure 1C,D). T+A@Glu‐NPs presented a uniformly distributed spherical structure as observed by transmission electron microscope (TEM) (Figure 1E). Furthermore, stability and GSH‐responsive release of T+A@Glu‐NPs were determined. As shown in Figure 1F–H and Figure S4 (Supporting Information), the nanoparticles were well stable in the simulated physiological environment and released rapidly and responsively in a 10 mM GSH environment. Finally, we used a single‐layer BBB model to initially assess the effect of T+A@Glu‐NPs penetration rate of the blood‐brain barrier compared with T+A@NPs in vitro, and found that after glucose modification, nanoparticles BBB penetration ratio increased by ≈15% (Figure 1I,J).

### 2.2 Validation of T+A@Glu‐NPs Targeting In Vitro and In Vivo

**Figure 2:**
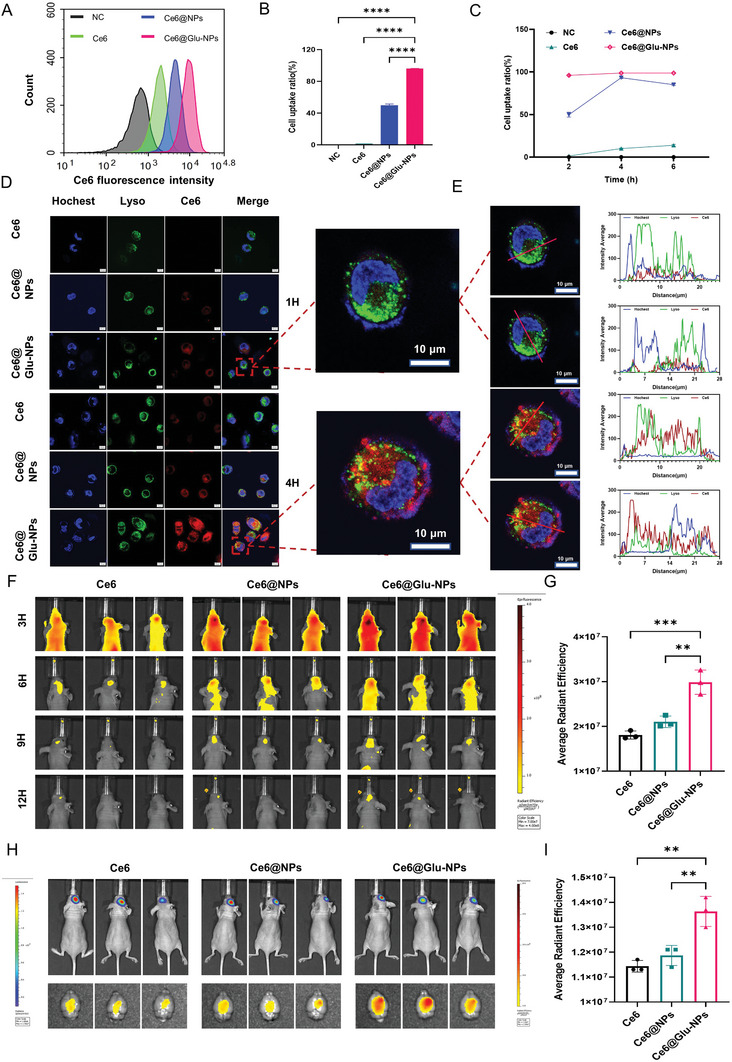
Enhanced targeting ability of T+A@Glu‐NPs in vitro and in vivo. A) FCM of Ce6 fluorescence intensity after LN229 incubated with Ce6, Ce6@NPs, Ce6@Glu‐NPs for 2 h. B) Statistical analysis of cell uptake rate after LN229 incubated with Ce6, Ce6@NPs, Ce6@Glu‐NPs for 2 h. C) Cellular uptake rate curves of different treatment groups. The untreated group was used as the control. D) The confocal images of the cells after LN229 incubated with Ce6, Ce6@NPs, Ce6@Glu‐NPs for 1 h and 4 h. E) The distribution of Ce6, Hochest33442 and LysoTracker Green DND‐26 in LN229 cells was examined using confocal imaging after incubated with Ce6@Glu‐NPs for 1h and 4 h. F) The distribution and changes in the Ce6 average radiant efficiency in the in vivo images of mice after tail vein injection of Ce6, Ce6@NPs, Ce6@Glu‐NPs at 3 h, 6 h, 9 h, and 12 h. G) Statistical analysis of the Ce6 average radiant efficiency in mice at 12 h in different treatment groups. H) The initial tumor size in vivo images of mice in each group and the distribution images of Ce6 average radiant efficiency in mouse brain tissues isolated at 24 h. I) Statistical analysis of Ce6 average radiant efficiency in mouse brain tissues isolated at 24 h. Data are presented as mean ± SD. No significant difference is marked with ns. *P < 0.05, **P < 0.01, ***P < 0.001 and ****P < 0.0001.

Chlorin e6 (Ce6) is auto‐fluorescent and can be used to probe the metabolic kinetics of drug‐carrying nanomaterials in vivo and the synthesis process of Ce6, Ce6@NPs, and Ce6@Glu‐NPs is presented in Supporting information. We first carried out cell uptake studies in vitro. As shown in **Figures** **2**A–C and S5 (Supporting Information), well‐grown glioma cells were treated with the same concentration of Ce6, Ce6@NPs, and Ce6@Glu‐NPs. We subsequently determined the uptake ratio of Ce6 by flow analysis. We found that the uptake ratio of Ce6@Glu‐NPs reached higher than 90% at 2 h, and it did not decrease significantly at 6 h. Whereas the free Ce6 group had a low level of uptake, the Ce6@NPs showed an intermediate rate of uptake but a faster decline. Thus, Glu‐modified nanoparticles both increased cellular uptake and prolonged the intracellular action time of the drugs. Besides, we found that the proportion of Glu‐PEG‐PCL in the nanoparticle shell affects the efficiency of drug uptake by the cells, as showed in Figure S6 (Supporting Information), the Ce6@Glu‐NPs L group took up more drug than the Ce6@Glu‐NPs H group. Second, we further examined nanoparticles travelling in glioma cells by confocal imaging and found that the concentration of Ce6@Glu‐NPs was the highest among the three groups in the cell at the same time point. Furthermore, we found that the Ce6@Glu‐NPs moved from the cytosol to lysosomes and then escaped into the nucleus (Figure 2D,E; Figure S7, Supporting Information). Finally, we constructed a glioma orthotopic model to verify the ability of Ce6@Glu‐NPs to target tumors in vivo. As shown in Figure 2F,G and Figure S8 (Supporting Information), the Ce6@Glu‐NPs accumulated in the brain tissue faster and more at the same time point and lasted for a longer time. Besides, the Ce6@Glu‐NPs concentrations remained highest in the isolated brain tissue at 24 h (Figure 2H,I; Figure S9, Supporting Information). Further, we separated normal brain tissue from glioma tissue and conducted fluorescence intensity measurements. We found that the average fluorescence intensity of Ce6 in normal brain tissue was essentially consistent, while in the glioma tissue, the fluorescence intensity of the Ce6@Glu‐NPs group was the highest. This further proves the dual‐targeting functionality of Ce6@Glu‐NPs (Figure S10, Supporting Information). Finally, we found that Ce6 was predominantly distributed in the liver in isolated organs, suggesting that it is metabolized through the liver (Figures S11 and S12, Supporting Information). All the results indicated that the Glu‐modified nanoparticles have superior targeting ability.

### 2.3 T+A@Glu‐NPs Induced Cells Proliferation Inhibition and Cells Apoptosis In Vitro

**Figure 3:**
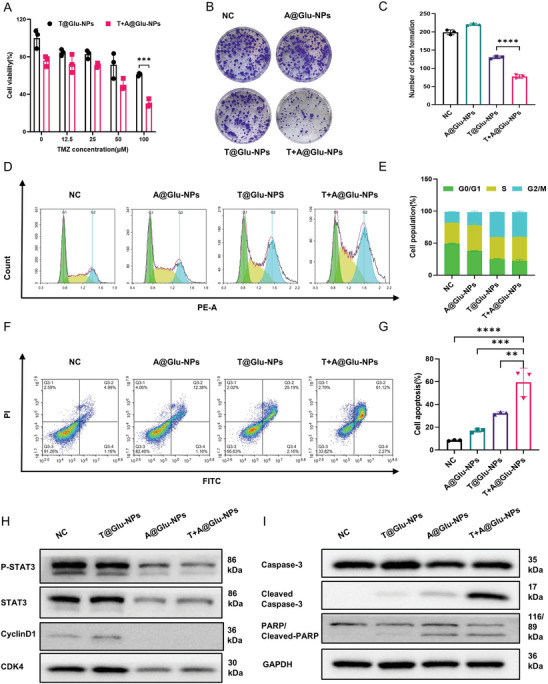
T+A@Glu‐NPs induced cells proliferation inhibition and cells apoptosis in vitro. A) Cell viabilities of GL261 cells treated with A@Glu‐NPs, T@Glu‐NPs and T+A@Glu‐NPs at different concentrations for 48 h (n = 3). The untreated group was used as a control. B) Representative images of colony formation of GL261 cells incubated with 100 ng mL^−1^ A@Glu‐NPs, 25 µM T@Glu‐NPs and T+A@Glu‐NPs. C) Quantification of colony formation (n = 3). D) GL261 cells cycle distribution determined by flow cytometry after treated with 100 ng mL^−1^ A@Glu‐NPs, 100 µM T@Glu‐NPs and T+A@Glu‐NPs for 48 h. E) Quantification of cell cycle distribution (n = 3). F) Flow cytometry detection of GL261 cells apoptosis after GL261 cells treated with 200 ng mL^−1^ A@Glu‐NPs, 75 µM T@Glu‐NPs and T+A@Glu‐NPs for 48 h. G) Statistical analysis of apoptosis cells (n = 3). H) Representative Western blot analysis of P‐STAT3, STAT3, Cyclin D1, CDK4 and GAPDH in GL261 cells treated with 100 ng mL^−1^ A@Glu‐NPs, 100 µM T@Glu‐NPs and T+A@Glu‐NPs for 48 h. GAPDH served as a loading control. I) Expressions of apoptosis related proteins (pro‐caspase 3, cleaved caspase 3, PARP) and the loading control GAPDH were determined by western blot analysis after GL261 cells treated with 100 ng mL^−1^ A@Glu‐NPs, 100 µM T@Glu‐NPs and T+A@Glu‐NPs for 48 h. J) Quantitative analysis of all the western blot results. Data are presented as mean ± SD. No significant difference is marked with ns. *P < 0.05, **P < 0.01, ***P < 0.001 and ****P < 0.0001.

We then assessed the cytotoxicity of T+A@Glu‐NPs against glioma cells through Cell Count Kit‐8 (CCK‐8) assay. The results showed the viability of cells treated with T+A@Glu‐NPs decreased more than the cells treated with T@Glu‐NPs only (**Figure** **3**A; Figures S13–S15, Supporting Information). Meanwhile, the inhibitory phenomenon was observed in colony formation assay. The clone numbers of cells incubated with T+A@Glu‐NPs were less than those of other groups, which were treated by T@Glu‐NPs, A@Glu‐NPs and NS respectively (Figure 3B, C; Figures S16 and S17, Supporting Information). We further evaluated the influence of the T+A@Glu‐NPs on the cell cycle in different glioma cell lines. We found that T+A@Glu‐NPs would significantly block the cell cycle of GL261 cells and U251 cells in G2/M phase, but there was a difference between U87cells and LN229 cells (Figure 3D,E; Figures S18–S20, Supporting Information). The results may be caused by the glioma heterogeneity. Western blot analysis was carried out to investigate the molecular mechanism of the T+A@Glu‐NPs for glioma suppression. As showed in Figure 3H, Figures S21 and S22 (Supporting Information), despite T@Glu‐NPs showing opposite trends in CDK4 expression in GL261 and LN229 cell lines, which could be due to the inherent differences between the two cells and the complex regulatory mechanisms of cyclins. The expression of cell cycle‐related proteins was downregulated obviously after glioma cells being incubated with T+A@Glu‐NPs for 48h, including cyclin D1 and CDK4. The results further confirmed the T+A@Glu‐NPs could block the cell cycle of glioma. Besides, we found the expression level of p‐STAT3 and STAT3 was also decreased, which were related to the cell proliferation in glioma.

To evaluate the effect of the chemotherapeutics on glioma cell apoptosis, FCM analysis and western blot assay were performed in the study. The T+A@Glu‐NPs showed strongest capacity to induce cell apoptosis in GL261 cells and U87 cells (Figure 3F,G; Figure S23, Supporting Information). Meanwhile, Western blot analysis results demonstrated that the T+A@Glu‐NPs induced cleavage of caspase 3 and PARP to activate the apoptosis pathway (Figure 3I; Figures S21 and S22, Supporting Information). All results showed that T+A@Glu‐NPs produce anti‐glioma efficacy by increasing cell apoptosis, blocking cell cycle and inhibiting cell proliferation.

### 2.4 T+A@Glu‐NPs Enhanced the Antitumor Efficacy of TMZ In Vivo

**Figure 4:**
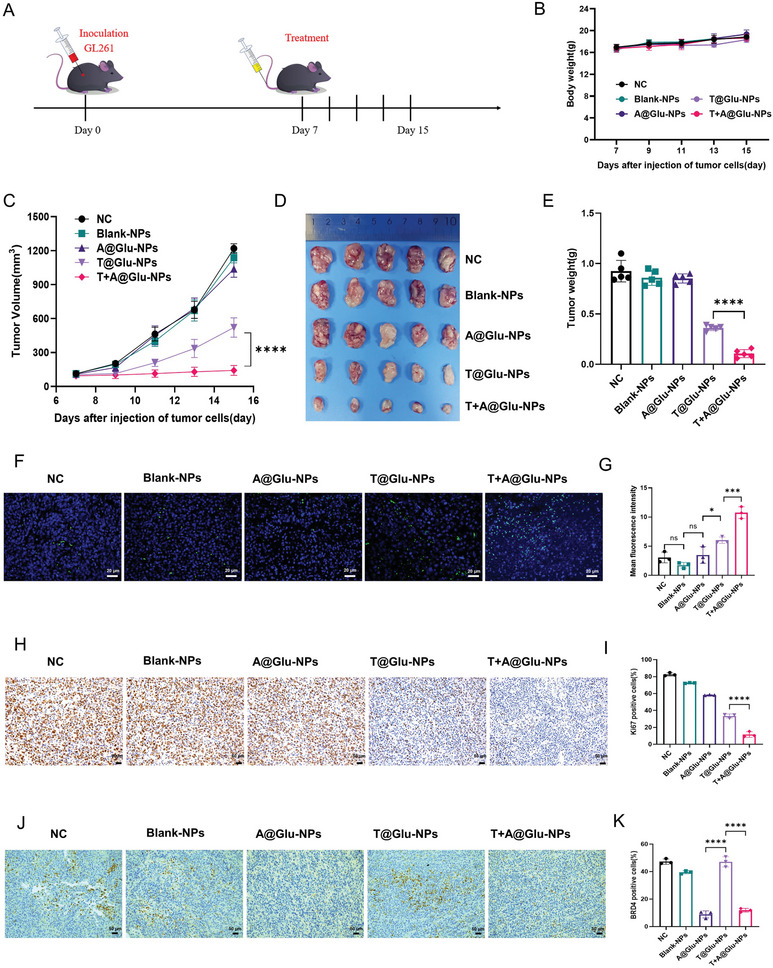
Antitumor effect of T+A@Glu‐NPs in GL261 glioma subcutaneous model. A) Schedule of experimental design in GL261 mouse subcutaneous model. B) Changes of body weight of mice in different group during treatment. C) The tumor group curve of the mouse subcutaneous model. D) The image of tumors from different groups. E) Quantification of tumors weight from different groups. F,G) TUNEL immunofluorescence staining images and statistical analysis of the GL261 mouse subcutaneous tumor. H,I) Ki67 staining images and statistical analysis of the GL261 mouse subcutaneous tumor. J,K) BRD4 immunohistochemical staining images and statistical analysis of the GL261 mouse subcutaneous tumor. Data are presented as mean ± SD. No significant difference is marked with ns. *P < 0.05, **P < 0.01, ***P < 0.001 and ****P < 0.0001.

**Figure 5:**
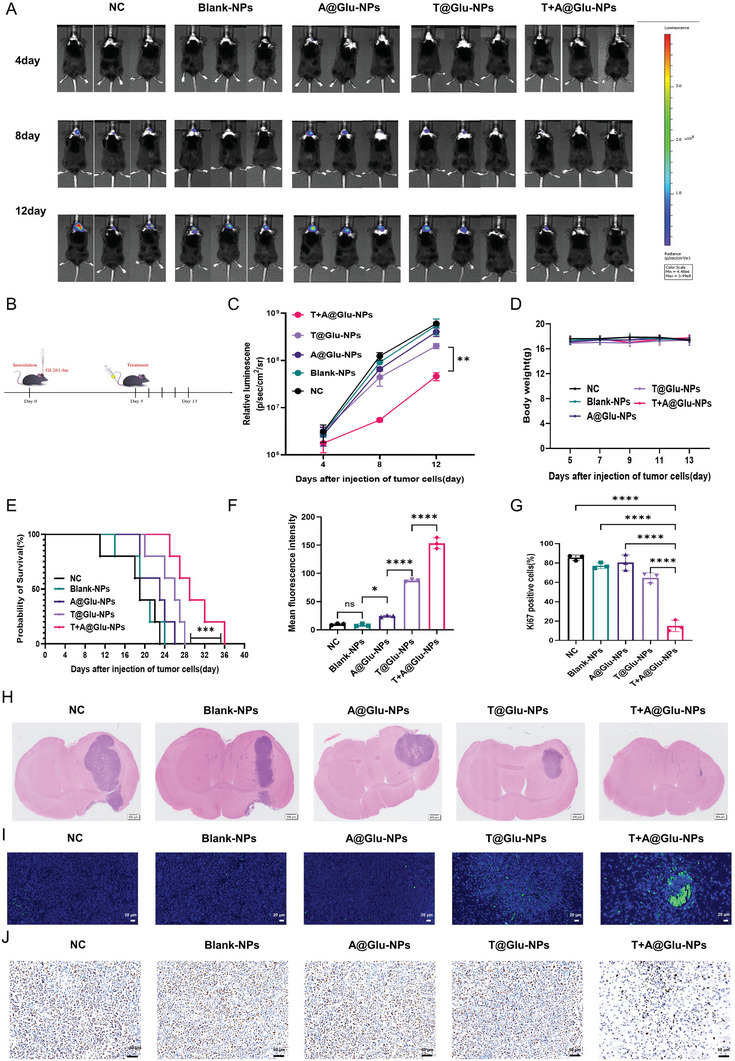
Antitumor effect of T+A@Glu‐NPs in GL261 glioma orthotopic model. A) In vivo imaging of GL261‐Luc tumor‐bearing mice from different groups at 4, 8 and 12 days after injection of tumor cells. B) Schedule of experimental design in GL261 mouse orthotopic model. C) The curve of relative luminescence in different treatment groups. D) Changes of body weight of mice in different group during treatment (n = 5). E) The overall survival curve of the GL261 mouse orthotopic model and the humane endpoint is the persistent discomfort of mouse, such as severe hunched posture, reduced activity, apathy, leg dragging, or weight loss of more than 20%. F) Statistical analysis of TUNEL immunofluorescence staining. G) statistical analysis of Ki67 staining. H) H&E staining images of the GL261 orthotopic tumor on day 13 after the inoculation of GL261 cells. I) TUNEL immunofluorescence staining images of the GL261 mouse orthotopic tumor. J) Ki67 staining images of the GL261 mouse subcutaneous tumor. Data are presented as mean ± SD. No significant difference is marked with ns. *P < 0.05, **P < 0.01, ***P < 0.001 and ****P < 0.0001.

**Figure 6:**
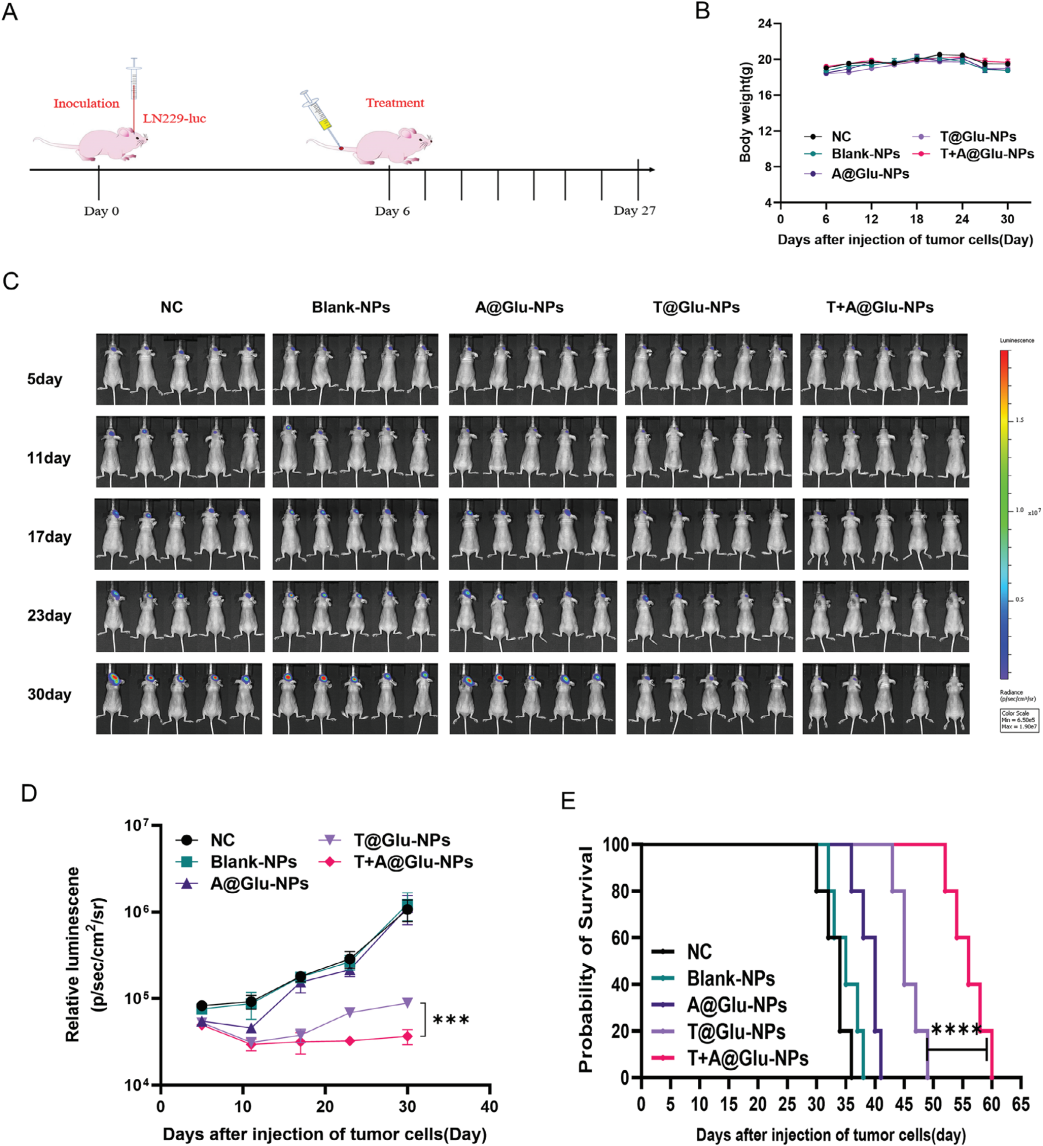
Antitumor effect of T+A@Glu‐NPs in LN229 glioma orthotopic model. A) Schedule of experimental design in mouse orthotopic model. B) Changes of body weight of mice in different group during treatment. C) In vivo imaging of LN229‐Luc tumor‐bearing mice from different groups at 5, 11, 17, 23, and 30 days after injection of tumor cells. D) The curve of relative luminescence in different treatment groups. E) The overall survival curve of the LN229 mouse orthotopic model. Data are presented as mean ± SD. No significant difference is marked with ns. *P < 0.05, **P < 0.01, ***P < 0.001 and ****P < 0.0001.

To evaluate the anti‐glioma effect of T+A@Glu‐NPs in vivo, we first established a GL261 subcutaneous tumor model. According to the schedule shown in **Figure** **4**A, the bearing tumor mice were randomly allocated into NC, Blank‐NPs, A@Glu‐NPs, T@Glu‐NPs or T+A@Glu‐NPs treatment group after the tumor volume grow up to ≈62.5 mm^3^, which were intravenously injected with different drugs once the other day for five times. During the treatment, the tumor volume and the mouse body weight before each injection were monitored. At the endpoint, both the T@Glu‐NPs and T+A@Glu‐NPs exhibited superior glioma inhibition capacity compared with the NC, Blank‐NPs and A@Glu‐NPs groups, and the T+A@Glu‐NPs attained a tumor suppression rate of 90%, while it was merely ≈50% in the T@Glu‐NPs group (Figure 4C–E). Moreover, the results of immunohistochemistry and immunofluorescence showed more significantly decrease in Ki67 index and increase in the mean fluorescence density of TUNEL apoptosis in the T+A@Glu‐NPs group compared to the T@Glu‐NPs group, suggesting that the T+A@Glu‐NPs exerted anti‐glioma effects by inhibiting tumor cell proliferation and inducing cells apoptosis (Figure 4F–I). GL261 glioma orthotopic mouse model was subsequently established to further assess the anti‐glioma efficacy of T+A@Glu‐NPs in vivo. The tumor‐bearing mice were randomly divided into five groups, including NC, Blank‐NPs, A@Glu‐NPs, T@Glu‐NPs, and T+A@Glu‐NPs, which received the corresponding drugs administered through the tail vein every two days, and in vivo monitoring of the tumor growth and records of the body weight changes of the mice were conducted (**Figure** **5**B). As shown in Figure 5A,C, there was no significant difference in tumor growth among NC, Blank‐NPs and A@Glu‐NPs groups, while tumor growth was inhibited in the T@Glu‐NPs group and the T+A@Glu‐NPs group, and the tumor suppression rate of T+A@Glu‐NPs group was ≈90%. After treatment, half of the mice were randomly selected for eye blood sampling in each group, and performed safety assessment and HE staining after stripping the mouse brain and other important organs. The results showed that the tumor volume in the brain tissue of the T+A@Glu‐NPs group was significantly reduced compared with that of A@Glu‐NPs and T@Glu‐NPs groups (Figure 5H), and the results of Ki67 and TUNEL staining were consistent with the subcutaneous glioma model (Figure 5F, G, I, and J). Moreover, the survival analysis showed that overall survival of the T+A@Glu‐NPs group was extended to 36 days and the overall survival of the T@Glu‐NPs group was only 28 days, which further verified the anti‐glioma efficacy of the T+A@Glu‐NPs was superior to that of T@Glu‐NPs (Figure 5E). Moreover, an orthotopic mouse model of human glioma LN229 in nude mice was established to further evaluate the anti‐glioma efficacy of T+A@Glu‐NPs in vivo. The experimental mice were randomized into 5 treatment groups, the dosing method and dosing concentration remained unchanged, but the dosing interval was changed to once every 3 days for a total of 8 doses (**Figure** **6**A). The in‐vivo imaging results showed that the T+A@Glu‐NPs group significantly inhibited tumor growth compared with the other groups, while the overall survival of mice in the T+A@Glu‐NPs was extended to 60 days from 49 days in the T@Glu‐NPs group (Figure 6C–E). Besides, there was no significant weight loss, organ damages and systemic toxicity observed in the experimental mice of the three glioma animal models, which suggested a reliable safety profile for the T+A@Glu‐NPs (Figures 4B, 5D, and 6B; Figures S24‐S27, Supporting Information). In summary, we could propose a conclusion that the T+A@Glu‐NPs has stronger anti‐glioma efficacy and is a promising and safe drug combination regimen.

### 2.5 Anti‐Glioma Mechanisms of T+A@NPs

**Figure 7:**
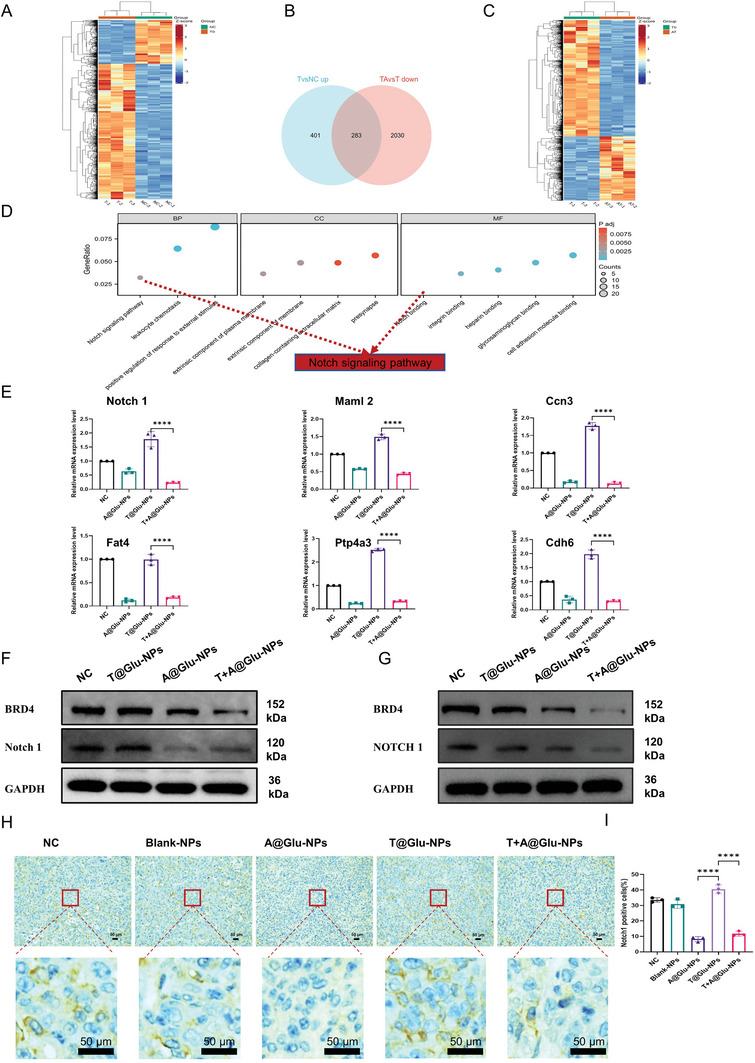
The mechanism of T+A@Glu‐NPs to reverse the TMZ resistance in glioma. A), B), and C) The RNA‐seq analysis and differential genes expression analysis of GL261 cells treated with 100 ng mL^−1^ A@Glu‐NPs, 100 µM T@Glu‐NPs, and T+A@Glu‐NPs for 48 h. The untreated group was as2333 a control. D) The enrichment analysis based on the RNA‐seq. E) Real‐time quantitative PCR analysis of genes related to the Notch1 signaling pathway. F,G) Western blot analysis of BRD4 and NOTCH1 after the GL261 cells treated with 100 ng mL^−1^ A@Glu‐NPs, 100 µM T@Glu‐NPs, and T+A@Glu‐NPs for 48 h. The untreated group was as a control. H,I) Notch1 immunohistochemical staining images and statistical analysis of the GL261 mouse subcutaneous tumor. Data are presented as mean ± SD. No significant difference is marked with ns. *P < 0.05, **P < 0.01, ***P < 0.001, and ****P < 0.0001.

With the aim to disclose the key molecules and pathways involved in the enhanced anti‐tumor effects of T+A@Glu‐NPs, the transcriptome sequencing analysis was performed to analyze changes in the expression levels of relevant genes after drug treatment of GL261 glioma cells for 48 h, and differential gene analysis and pathway enrichment analysis were sequentially implemented to find possible key genes and pathways. As shown in **Figure** **7**A–D, Notch1 gene and related pathways were upregulated in T@Glu‐NPs group, while were downregulated significantly in the T+A@Glu‐NPs group, which indicated Notch1 gene and related pathways maybe play a critical role in glioma TMZ resistance. To further verify the hypothesis, the expression levels of the Notch1 gene and related pathway genes were detected by real time qPCR. The results demonstrated that Notch1 and notch pathway related genes were up‐regulated after T@Glu‐NPs treatment and could be down‐regulated after A@Glu‐NPs and T+A@Glu‐NPs treatment, including Maml2, Ccn3, Fat4, Ptp4a3 and Cdh6 (Figure 7E). Based on the above results, it is speculated that Notch1 gene might play a key role in glioma TMZ resistance, while ARV‐825 could degrade BRD4 protein to reverse glioma TMZ resistance by modulating Notch1 pathway related genes expression. Thereupon, western blot analysis was further performed to confer the correlation between BRD4 and Notch1 protein expression after glioma cells were treated for 48 h. As shown in Figure 7F,G, the expression of BRD4 and Notch1 was consistent in the same therapy group and was slightly upregulated in the T@Glu‐NPs group, while was significantly downregulated in the the A@Glu‐NPs and T+A@Glu‐NPs groups. The results were basically consistent with the previous transcriptome sequencing analysis and real time qPCR analysis results, substantiating the positive correlation between Notch1 gene and BRD4 protein in glioma. To further determine this point, the protein expression levels in tumor samples from animal models were probed by immunohistochemical techniques and found that the proportions of BRD4‐ and Notch1‐positive cells were slightly increased in T@Glu‐NPs and significantly decreased in tumor samples from both A@Glu‐NPs and T+A@Glu‐NPs (Figures 4J,K, and 7H,I). Therefore, we suggested that Notch1 may mediate glioma TMZ resistance, and T+A@Glu‐NPs can downregulate Notch1 by degrading BRD4 protein, thereby reversing glioma TMZ resistance.

### 2.6 Knocking out Notch1 Gene Enhanced the Anti‐Glioma Efficacy of TMZ In Vitro and In Vivo

**Figure 8:**
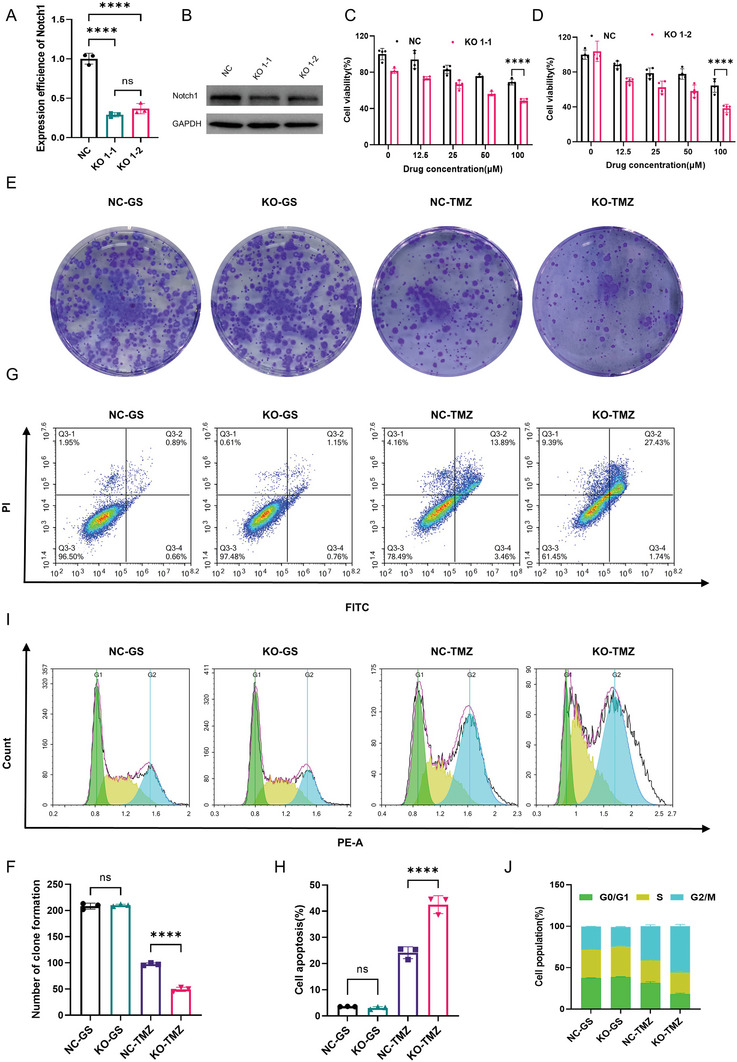
Knocking out Notch1 gene enhanced the anti‐tumor efficacy of TMZ in vitro. A) The qPCR of two Notch1 gene knocked out clones. (B) Western blot analysis of Notch1. GAPDH served as a loading control. C) Cell viabilities of clone 1‐1 in GL261‐KO cells treated with T@Glu‐NPs at different concentrations for 48 h. D) Cell viabilities of clone 1–2 in GL261‐KO cells treated with T@Glu‐NPs at different concentrations for 48 h. E,F) Representative images of colony formation of GL261(NC/KO) cells incubated with 25 µM TMZ/NPs and quantification of colony formation. G,H) Flow cytometry detection of GL261(NC/KO) cells apoptosis after different treatments for 48 h and statistical analysis of apoptosis cells. I,J) GL261 (NC/KO) cells cycle distribution determined by flow cytometry after treatment with 100 µM T@Glu‐NPs for 48 h and quantification of cell cycle distribution. Data are presented as mean ± SD. No significant difference is marked with ns. *P < 0.05, **P < 0.01, ***P < 0.001 and ****P < 0.0001.

Many studies have shown that Notch1 is involved in the tumor growth, metastasis, drug resistance and recurrence of a variety of tumors, especially its close relationship with glioma stem cells.^[^ ^39^, ^40^, ^41^, ^42^, ^43^ ^]^ In this study, we speculated that Notch1 could be a key molecule in the TMZ resistance of gliomas and subsequently constructed Notch1 knockout GL261 cell lines. As shown in **Figure** **8**A,B, there were two Notch1 knockout GL261 cell lines, KO1‐1 and KO1‐2, which had been verified by quantitative PCR and Western blot analysis. After that, CCK‐8 was performed and it was found the GL261/KO1‐1 and GL261/KO1‐2 were more sensitive to TMZ after Notch1 was knockout compared to the GL261/NC cell line (Figure 8C,D). Besides, the colony formation assay results showed that knockout of Notch1 would enhance proliferation inhibition of gliomas by TMZ (Figure 8E,F). Moreover, flow cycle and apoptosis analysis were further used to explore whether knockout of the Notch1 gene enhanced cycle block and increased cell apoptosis of glioma by TMZ. We found that knockout of Notch1 did enhance the TMZ‐induced cycle block in G2/M phase and induced an increase in apoptosis by TMZ (Figure 8G–J). Furthermore, NOTCH1 knockout human‐derived glioma cell lines were also performed. As shown in Figures S28 and S29 (Supporting Information), both the LN229 NOTCH1 gene knockout cell lines and the U251 NOTCH1 gene knockout cell lines were again sensitive to TMZ compared the normal LN229 and U251 cell lines. In short, knockdown of Notch1 gene would make glioma sensitized to TMZ again in vitro.

**Figure 9:**
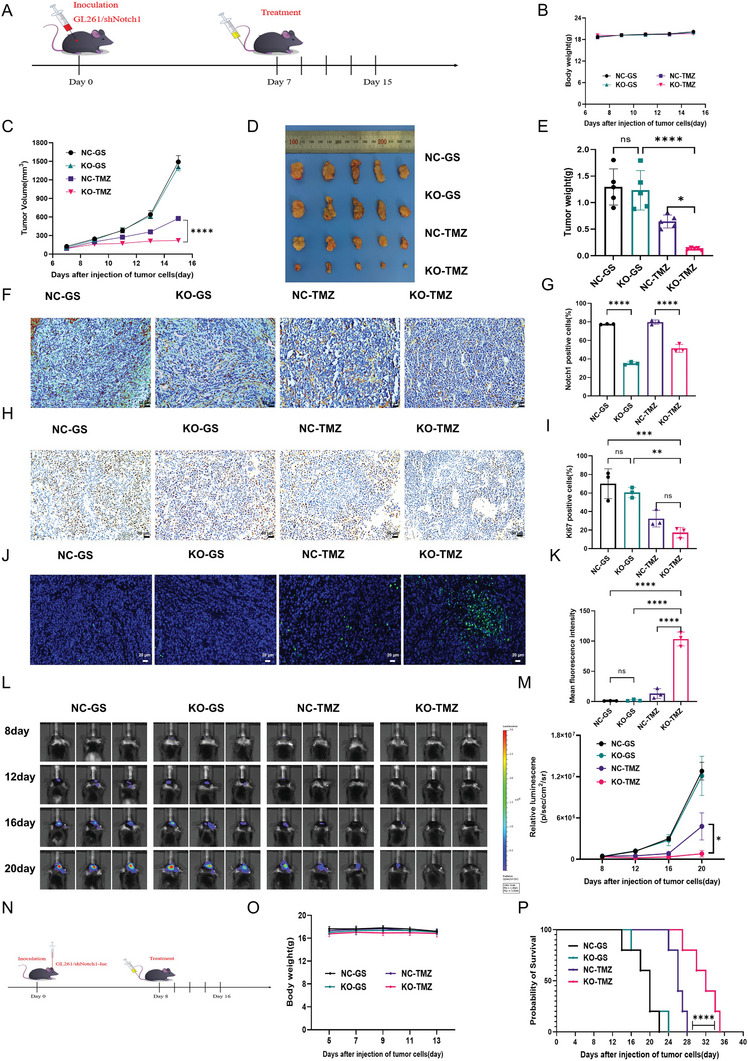
Knocking out Notch1 gene enhanced the anti‐tumor efficacy of TMZ in GL261 subcutaneous and orthotopic mouse model. A) Schedule of experimental design in GL26 (NC/KO) mouse subcutaneous model. B) Changes of body weight of mice in different group during treatment. C) The tumor group curve of the mouse subcutaneous model. D) The image of tumors from different groups. E) Quantification of tumors weight from different groups. F,G) BRD4 immunohistochemical staining images and statistical analysis of the GL261 (NC/KO) mouse subcutaneous tumor. H,I) Ki67 staining images and statistical analysis of the GL261 (NC/KO) mouse subcutaneous tumor. J,K) TUNEL immunofluorescence staining images and statistical analysis of the GL261 (NC/KO) mouse subcutaneous tumor. L) In vivo imaging of GL261‐Luc tumor‐bearing mice from different groups at 4, 8, and 12 days after injection of tumor cells. M) The curve of relative luminescence in different treatment groups. N) Schedule of experimental design in GL261‐Luc (NC/KO) mouse orthotopic model. O) Changes of body weight of mice in different group during treatment. P) The overall survival curve of the GL261‐Luc (NC/KO) mouse orthotopic model. Data are presented as mean ± SD. No significant difference is marked with ns. *P < 0.05, **P < 0.01, ***P < 0.001, and ****P < 0.0001.

Considering the complex effects of the internal environment and tumor microenvironment, the GL261/KO1‐2 and GL261/NC cell lines were inoculated into mice subcutaneously to construct both NOTCH1 KO and NOTCH1 NC mice models of subcutaneous tumor, respectively. As shown in **Figure** **9**A, mice were randomly divided into four groups, NC‐GS, NC‐TMZ, KO‐GS, and KO‐TMZ, and were given the corresponding drug treatments. When the subcutaneous tumor volume of mice in the NC‐GS group grew to ≈1500 mm^3^, the experiment was stopped and the subcutaneous tumor was peeled off. We found that there was no significant difference in the growth curves of gliomas, the weight and volume of isolated tumors between the NC‐GS and KO‐GS groups, while tumor growth was significantly slower in the KO‐TMZ group than that in the NC‐TMZ group (Figure 9C–E). And the knockout of Notch1 gene increased TMZ‐induced tumor proliferation inhibition and tumor cell apoptosis. Moreover, the results of Ki67 and TUNEL staining were consistent with previous results (Figure 9F–K). Subsequently, the GL261/KO1‐2 and GL261/NC cell lines were transfected with plasmids stably expressing luciferase to construct the new cell lines, GL261/KO1‐2‐Luc and GL261/NC‐Luc. Then the cells were inoculated into mouse brain to construct two kinds of orthotopic glioma mice, NC‐Luc and KO‐Luc (Figure 9N). The mice were randomly divided into NC‐GS, NC‐TMZ, KO‐GS and KO‐TMZ groups according to the results of the first live imaging, and were injected appropriate drugs (Figure 9N). During the treatment, small animal live imaging was performed every four days and all the death of the mice in the KO‐TMZ group as the endpoint of the survival observation. The results at the end of the experiment showed that there was no significant difference in the change of in situ tumor fluorescence intensity curves between the NC‐GS and KO‐GS groups, and tumor growth was significantly slower in the KO‐TMZ mice than that in the NC‐TMZ group (Figure 9L,M), and the overall survival of KO‐TMZ mice was extended to 35 days, while it was only 28 days in the NC‐TMZ group (Figure 9P). Besides, there was no weight loss in all groups (Figure 9B,O). All in all, we concluded that knockout of the Notch1 gene enhances the glioma therapeutic effectiveness of TMZ and further confirmed that the Notch1 gene is a key molecule in the reversal of TMZ resistance in glioma.

### 2.7 Mechanisms of T+A@Glu‐NPs to Modulate Notch1 Expression to Enhance Anti‐Glioma Efficacy of TMZ

**Figure 10:**
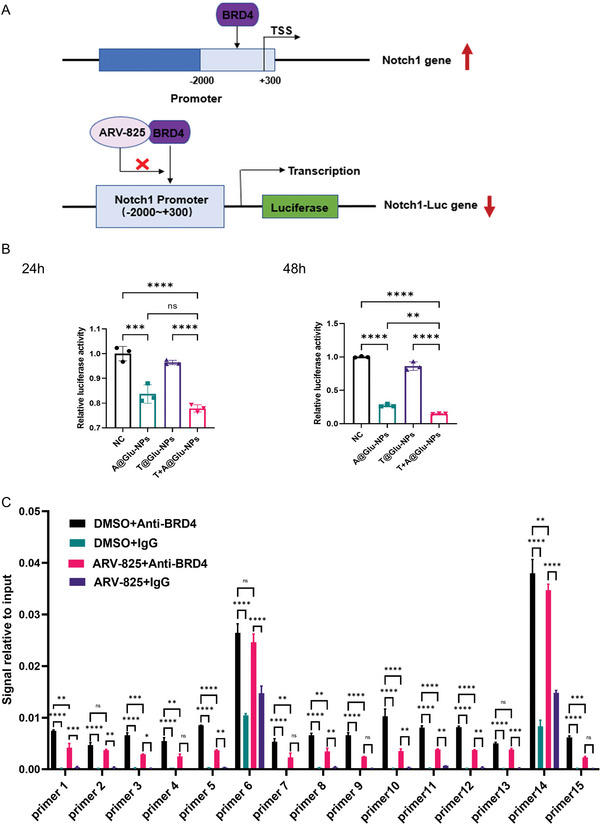
Mechanisms of T+A@Glu‐NPs modulate Notch1 expression to enhanced anti‐glioma efficacy of TMZ. A) The diagram of BRD4 acting on the Notch1 gene promoter and ARV‐825 reducing luciferase gene transcription initiated by the Notch1 promoter through inhibiting BRD4. B) Relative Notch1 luciferase reporter activity in GL261 cells treated with 100 ng mL^−1^ A@Glu‐NPs, 100 µM T@Glu‐NPs and T+A@Glu‐NPs for 48 h. The untreated group was as a control. C) ChIP‐qPCR analysis of Notch1 promoter in DMSO or 100 ng mL^−1^ A@Glu‐NPs treated GL261 cells for 48 h using IgG or anti‐BRD4. Data are presented as mean ± SD. No significant difference is marked with ns. *P < 0.05, **P < 0.01, ***P < 0.001, and ****P < 0.0001.

As an important epigenetic regulation factor, BRD4 acts as a transcriptional bridging platform and is able to regulate the transcription of a variety of genes. Besides, previous studies have shown that Notch1 expression is positively correlated with BRD4 expression.^[^ ^44^, ^45^ ^]^ Therefore, we first performed a dual luciferase reporter gene assay to explore whether BRD4 was involved in the transcriptional regulation of NOTCH1 genes and whether T+A@Glu‐NPs could degrade BRD4 protein to downregulate Notch1 expression. As shown in **Figure** **10**A,B, the intensity of firefly luciferase in T+A@Glu‐NPs treated cells was significantly reduced compared to that in the control group, indicating a reduction in the transcription of the Notch1 gene, which verified that T+A@Glu‐NPs might reduce the activation of the Notch1 promoter by degrading BRD4 protein, thereby reducing luciferase expression. So, we could conclude that BRD4 protein did regulate NOTCH1 gene expression. To further identify the promoter binding region of the BRD4 protein on the Notch1 gene, we designed 15 pairs of quantitative PCR primers for the NOTCH1 gene promoter sequence and set up a control group and an ARV‐825 drug‐treated group. After 48 h, the DNA bound to the BRD4 protein in each group was precipitated and purified according to the Chromatin Immunoprecipitation Kit, after which quantitative PCR was performed to verify the results. As shown in Figure 10C, BRD4 protein bound to the promoter region of the Notch1 gene and was involved in the transcriptional regulation of this gene. In another word, ARV‐825 could degrade BRD4 protein thereby inhibiting the transcription of the Notch1 gene.

## 3 Discussion

Currently, TMZ resistance in gliomas remains a significant challenge in clinical glioma chemotherapy, so there is an increased urgency for new drug combination therapies. A multitude of small molecule inhibitors of the BET family proteins have been employed to enhance the synergy of tumor chemotherapy, and these studies have demonstrated that the inhibition of BET proteins can indeed sensitize tumors to chemotherapy and extend survival in mice.^[^ ^46^, ^47^, ^48^ ^]^ However, these small molecule inhibitors are more prone to off‐target effects and systemic toxicity due to the necessity for higher doses. In contrast, the PROTAC technology ubiquitinates target proteins by linking the target protein ligand to a ubiquitin E3 ligase, and then efficiently and specifically degrades the target protein with the assistance of the ubiquitin proteasome system, achieving sustained degradation at very low concentrations.^[^ ^49^, ^50^ ^]^ Consequently, various PROTAC formulations have been applied in tumor chemotherapy, yielding improved efficacy. And the ARV‐825, as a BRD4 PROTAC, requires minimal doses to efficiently degrade target proteins and exhibits reduced susceptibility to drug resistance in single‐agent glioma treatments. So, in this study, we propose for the first time the use of ARV‐825 as a therapeutic adjuvant for TMZ in gliomas.

Furthermore, to overcome the challenges of poor systemic drug targeting and low blood‐brain barrier (BBB) permeability, we designed glucose‐modified nano micelles, Glu‐PEG‐PCL, to construct a drug delivery platform. This platform ingeniously exploits the glucose transporter, which is highly expressed in both the cerebral vascular endothelium and glioma cell membranes, to deliver drugs in a targeted and efficient manner. Additionally, to ensure effective drug release within glioma cells, we incorporated a disulfide bond into the nanomaterials, which can be responsively cleaved in the high GSH environment of the glioma periplasm, enabling targeted drug release.^[^ ^51^, ^52^, ^53^ ^]^ The results indicated that the modified glucose molecules facilitated dual targeting of T+A@Glu‐NPs to the BBB and gliomas in a “two birds with one stone” style, and the GSH‐responsive crosslinker containing disulfide bonds ensured “directional blasting” cleavage of the nanoparticles to release TMZ and ARV‐825 in the high GSH environment of glioma cells. Moreover, the safety evaluation in mice revealed no significant toxic side effects in terms of blood biochemistry and vital organs, as well as no abnormal weight loss in mice during treatment, demonstrating the reliable safety of this delivery system as a drug delivery platform. All results suggest that T+A@Glu‐NP is an efficient and promising drug delivery platform for glioma chemotherapy.

Ultimately, T+A@Glu‐NPs significantly inhibited the glioma progression and prolonged the overall survival of the tumor bearing mice through inducing cell apoptosis, inhibiting cell proliferation and blocking cell cycle. More importantly, we found that the expression levels of Notch1 were positively correlated with BRD4 proteins in Western blot analysis and immunohistochemistry results of in vivo tumor samples. Furthermore, T+A@Glu‐NPs were able to significantly downregulate the expression of Notch1, as revealed by transcriptome sequencing. Subsequent dual‐luciferase reporter assays and ChIP‐PCR experiments conclusively verified that BRD4 protein could bind to the Notch1 promoter to regulate its transcription. Meanwhile, Notch1 pathway‐related genes, including Maml2, Ccn3, Fat4, Cdh6, and Ptp4a3, exhibited similar expression trends and may be intimately linked to glioma development. Additionally, knockout of the Notch1 gene enhanced the efficacy of TMZ in gliomas and the survival benefit of mice. We therefore conclude that ARV‐825 can degrade BRD4 protein to downregulate the transcription of the Notch1 gene, reversing TMZ resistance in gliomas. It is somewhat regrettable that we did not explore the downstream targets of the Notch pathway, which would be a direction for future research.

In brief, the T+A@Glu‐NPs is a novel and potential combined strategy for glioma chemotherapy and the sensitization of glioma chemotherapy by T+A@Glu‐NPs through modulation of Notch1 is a novel and main mechanism, offering a new direction for the development of new glioma chemotherapeutic regimens and providing more options for clinical glioma treatment.

## 4 Conclusion

In summary, the Glu‐modified nanoparticles we constructed effectively penetrated the blood‐brain barrier and targeted gliomas, enabling precise drug delivery. Concurrently, T+A@Glu‐NPs have demonstrated strong efficacy against gliomas in both in vivo and in vitro studies. Furthermore, we have elucidated that T+A@Glu‐NPs exert their effects by modulating Notch1 expression. In conclusion, the T+A@Glu‐NPs we developed represent a novel and promising strategy for glioma chemotherapy. The elucidation of their mechanism of action could broaden the scope of drug combination regimens and offer valuable insights for clinical practice.

## 5 Experimental Section

### Preparation and Characterization of T+A@Glu‐NPs

The T+A@Glu‐NPs were prepared using a nano‐precipitation method. 2 mg TMZ, 2 mg ARV825, 30 mg PEG‐SS‐PCL, and 10 mg Glu‐PEG‐PCL were dissolved in 500 µL DMSO. The solution was then quickly added to ten times the volume of water and ultrasonicated for an additional 10–15 min. The organic phase and free drug were removed by ultrafiltration centrifugation to obtain T+A@Glu‐NPs. Unmodified nanoparticles were prepared by the same method.

Dynamic Light Scattering (Brookhaven Instruments, US) was initially employed to determine the size and polydispersity index (PDI) of T+A@Glu‐NPs. Malvern particle sizer (Malvern Nano ZS90 ZEN3690) was used for measuring the zeta potential of T+A@Glu‐NPs. Subsequently, Transmission Electron Microscopy (TEM, JEM‐2100Plus, Japan) was utilized to observe the dimensional and morphological characteristics of T+A@Glu‐NPs. In order to assess the stability of T+A@Glu‐NPs, the nanoparticles were diluted in pure water at 4°C and in 10% FBS at 37°C to simulate storage conditions and conditions in plasma, respectively. The particle size and PDI of T+A@Glu‐NPs were measured at intervals of 0, 1, 2, 4, 6, 8, 24, 48, 96, and 144 h, with each measurement replicated three times under the same conditions. Moreover, to evaluate the reduction responsiveness of the nanoparticles, T+A@Glu‐NPs were diluted in three different PBS buffers at pH 7.4, each containing 10 mM GSH, 0.1 mM GSH, and 0 mM GSH. The samples were then incubated at 37°C on a shaker at 100 rpm to ensure consistent concentration and distribution of the drug and GSH. The size and PDI of T+A@Glu‐NPs were measured at 0, 1, 2, 4, 6, 8, and 12 h post‐incubation. Meanwhile, TEM was utilized again to observe the changes on dimensional and morphological characteristics of T+A@Glu‐NPs after incubated in 10 mM GSH buffer for 8 h.

### Cell Uptake Assay

GL261 or LN229 cells were digested with trypsin and inoculated into 12‐well plates at a certain density (2 × 10^4^ per well for GL261 and 5× 10^4^ per well for LN229), and were incubated overnight at 37°C in an incubator. The next day, the GL261 or LN229 cells were incubated with Ce6, Ce6@NPs and Ce6@Glu‐NPs at a concentration of 2 µg mL^−1^. Afterwards, samples were collected for flow cytometry to determine intracellular Ce6 fluorescence intensity at 1 h, 2 h, and 4 h time points, respectively.

### Confocal Imaging

GL261 or LN229 cells were digested with trypsin and planted into confocal dishes at a certain density (1× 10^4^ per well for GL261 and 1.2× 10^4^ per well for LN229), and were incubated overnight at 37°C in an incubator. The next day, the GL261 or LN229 cells were incubated with Ce6, Ce6@NPs and Ce6@Glu‐NPs at a concentration of 20 µg mL^−1^. At 1 h and 4 h timepoints, samples were taken to terminate the incubation, and confocal imaging was performed after Hoechst 33342 and LysoTracker Green DND‐26 staining.

### Cell Viability Assay

GL261, LN229, U251, and U87 cells were digested with trypsin and inoculated into 96‐well plates at a certain density (2 × 10^3^ per well for GL261, 4 × 10^3^ per well for LN229, 8 × 10^3^ per well for U251 and U87), and were incubated overnight at 37°C in an incubator. The next day, the GL261 or LN229 cells were incubated with different concentrations of A@Glu‐NPs, T@Glu‐NPs and T+A@Glu‐NPs for 48 h. The untreated group served as a negative control. Sequentially, 20 µL of CCK‐8 was added to each well and incubated for another 1–2 h. The absorbance value (OD:450 nm) was determined by enzyme marker.

### FCM Detection of Cells Apoptosis

GL261 or LN229 cells were digested with trypsin and inoculated into 6‐well plates at a density of 1×10^5^ per well, and were incubated overnight in the incubator. The next day, GL261 or LN229 cells were incubated with certain concentrations of A@Glu‐NPs, T@Glu‐NPs and T+A@Glu‐NPs for 48 h. The untreated group served as a negative control. The supernatants were recovered and washed once with pre‐cooled PBS at 4°C, then 500 µL EDTA‐free trypsin digestion was added to each well. The digested glioma cells were collected into flow‐through tubes, centrifuged, resuspended, and washed twice with pre‐cooled PBS. Finally, 100 µL prepared staining solution (containing 5 µL annexin‐FITC and 5 µL PI per 100 µL) was added to each sample tube, and incubated at room temperature and protected from light for 15 min. 400 µL 1× Binding buffer was added to each tube and gently resuspended before flow apoptosis assay was performed using a flow cytometer.

### FCM Analysis of Cell Cycle

GL261 or LN229 cells were digested with trypsin, inoculated into 6‐well plates at a density of 1×10^5^ per well, and incubated overnight in the incubator. The double‐free medium was changed 12 h before drug addition. After that, GL261 or LN229 cells were incubated with certain concentrations of A@Glu‐NPs, T@Glu‐NPs and T+A@Glu‐NPs for 48 h. The untreated group served as a negative control. The cells were washed once with pre‐cooled PBS at 4°C, and 500 µL trypsin was added to each well for digestion. The digested glioma cells were collected into flow tubes, centrifuged, resuspended, and washed twice with pre‐cooled PBS. Finally, the cells were resuspended in 2 mL of pre‐cooled 75% ethanol and fixed at 4°C for 24 h. The fixed samples were added with the configured staining solution per tube and incubated at room temperature away from light for 30 min. Sequentially, the flow cycle analysis was performed at a low speed.

### Colony Formation Assay

GL261 or LN229 cells were digested with trypsin and inoculated into 6‐well plates at a density of 500–1000 per well, and were incubated overnight in the incubator. After that, GL261 or LN229 cells were incubated with certain concentrations of A@Glu‐NPs, T@Glu‐NPs and T+A@Glu‐NPs for 72 h. The untreated group served as a negative control. The drug‐containing medium was changed every 3 days until the cell number of single clone formation reached ≈50–60. Removed the supernatant, washed with PBS once, and fixed the cells with 1mL of 4% paraformaldehyde tissue fixative per well for 30–60 min. Then removed the fixative, washed with PBS again, and stain with 500 µL of 0.1% crystal violet staining solution per well for 10–20 min. Recycled the staining solution, washed the plates with PBS for 3 times or more, air dry, and take pictures.

### shRNA Knockdown Experiment

First, Notch1 shRNA primer sequences were first designed and then recombined with PLKO.1 plasmid to construct a new knockout plasmid. The empty vector as negative control. Then, viral supernatants containing large amounts of the target plasmid were obtained after plasmid transformation, single clone picking, sequencing and identification, and viral packaging. Sequentially, well‐grown GL261 cells were inoculated into 6‐well plates at 2×10^4^ per well, cultured overnight, and added the virus supernatant for infection. After 48 h of infection, puromycin at concentration of 1 µg mL^−1^ was added to screen cells for 48–72 h. Finally, the screened GL261 cells were re‐laid in plates for subsequent WB and qPCR experiments to verify the knockdown efficiency.

### Dual‐Luciferase Reporter Assay

GL261 cells were digested with trypsin, inoculated into 12‐well plates at a density of 5×10^4^ per well and cultured overnight. Double‐free medium was changed before transfection for 2–3 h. The prepared system containing PRL‐SV40 and Notch1‐Luc was added to the well plates according to the amount of 1 µg PRL‐SV40+1 µg Notch1‐Luc+2 µL Lipofectamine 2000 per well. After 4–6 h, the complete medium containing the drugs was switched, and the incubator was continued to incubate for 48 h. Finally, according to the Meilunbio Firefly & Renilla Luciferase Reporter Assay Kit to determine luciferase activity.

### Chromatin Immunoprecipitation (ChIP) Assay

ChIP assays were performed via the ChIP Assay Kit (Cell Signaling Technology, USA) according to the manufacturer's instructions. In brief, GL261 cells were incubated with 100 ng mL^−1^ A@Glu‐NPs and equal volume of DMSO respectively for 48 h, followed by fixing with formaldehyde to crosslink targeted proteins and DNA to form chromatin complexes. After breaking cells with ultrasonic, the chromatin complexes were immunoprecipitated via anti‐BRD4 antibody (Cell Signaling Technology, USA) or negative control IgG, and subsequently the protein G magnetic beads were used to capture the antibody‐chromatin complexes formed in front, followed by elution and decrosslinking. Finally, the purified DNA samples were detected by qRT‐PCR. The primers are listed in Supporting Information Table.

### Subcutaneous Xenograft Model

GL261‐WT and GL261‐KO cells were subcutaneously inoculated into C57BL/6 female mice at 5×10^6^ cells each mouse. When the tumors grew to 5 mm × 5 mm, the mice were randomly divided into four groups: (A) NC‐GS, (B) NC‐TMZ, (C) KO‐GS, and (D) KO‐TMZ. Each mouse was injected with 200 µL of the drug solution in the tail vein, and the dose of T@Glu‐NPs was 10 mg kg^−1^ body weight. The drug was administered every two days for a total of four times, and the changes in tumor volume and body weight of the mice were recorded at the same time. When the tumor volume reached 15 mm×15 mm, the mice were sacrificed and the tumors were weighted and photographed, and then the tumor tissues were sent for immunohistochemistry‐related sections.

### Glioma Orthotopic Xenograft Model

GL261‐luc or LN229‐luc cells were digested and washed twice with double‐free medium. C57BL/6 mice or nude mice were anesthetized and the hair on the head of the mice was removed. Then, a certain concentration cells (2×10^5^ cells for GL261‐luc, 5×10^5^ cells for LN229‐luc) were injected into mice brain. 4–5 days after inoculation, the mice were randomly divided into five groups according to fluorescence intensity: (1) NC; (2) Blank‐NPs; (3) A@Glu‐NPs; (4) T@Glu‐NPs; and (5) T+A@Glu‐NPs. The drugs were administered every 2 days for a total of 5 times in GL261‐luc orthotopic model and every 3 days for a total of 8 times in LN229‐luc orthotopic model. During treatment, tumor growth was monitored by in vivo imaging techniques and the body weight of the mice were recorded. At the end, blood samples serums were collected and prepared for the blood biochemistry test, and the whole brain and important organs were separated, fixed in 4% paraformaldehyde and the brain tumor tissues and vital organs were sent for immunohistochemistry and immunofluorescence staining. All animal experiments were approved by the Animal Experimentation Ethics Committee of the State Key Laboratory of Biotherapeutics (SKLB), Sichuan University, and were conducted in accordance with the Guidelines for the Care and Use of Institutional Animals.

### Statistical Analysis

In this study, data were presented as mean ± standard deviation of measurement (SD). The statistical differences of data were analyzed with the unpaired two‐tailed Student t test for two groups comparison and ordinary one‐way analysis of variance for multiple groups comparison in GraphPad Prism 9.0 software. The P value of more than 0.05 indicated no significant difference and was marked with ns. P value of less than 0.05 was considered statistically significant and were presented as *P < 0.05, **P < 0.01, ***P < 0.001 and ****P < 0.0001.

## Conflict of Interest

The authors declare no conflict of interest.

## Supporting information



Supporting Information

## Data Availability

Research data are not shared.
