# Supplementary material for: BRD4 Degradation Enhanced Glioma Sensitivity to Temozolomide by Regulating Notch1 via Glu‐Modified GSH‐Responsive Nanoparticles
Source: Adv Sci (Weinh). 2024 Nov 15;11(48):2409753. doi: 10.1002/advs.202409753 (PMC11672279; doi:10.1002/advs.202409753)
Supplement: Supplementary file 1 — Supporting Information [file ADVS-11-2409753-s001.docx]

**Supporting Information for**

**BRD4 degradation enhanced glioma sensitivity to temozolomide by regulating Notch1 via Glu-modified GSH-responsive nanoparticles**

Linbin Yi^a#^, Zhenyu Zhang^b#^, Wenjie Zhou^c,d#^,Yunchu Zhang^a^, Yuzhu Hu^a^, Anjie Guo^a^, Yongzhong Cheng^a^, Zhiyong Qian^a^, Peizhi Zhou^a*^, Xiang Gao^a*^

^a^Department of Neurosurgery and Institute of Neurosurgery, State Key Laboratory of Biotherapy and Cancer Center, West China Hospital, West China Medical School, Sichuan University and Collaborative Innovation Center for Biotherapy, Chengdu 610041, China.

^b^Department of Plastic and Burn Surgery, West China School of Medicine, West China Hospital, Sichuan University, Chengdu, 610041, China.

^c^Department of Laboratory Medicine, West China Second University Hospital, Sichuan University, Chengdu, China

^d^Key Laboratory of Birth Defects and Related Diseases of Women and Children (Sichuan University), Ministry of Education, China

* Corresponding author: Peizhi Zhou and Xiang Gao

E-mail: peizhizhouns@126.com and xianggao@scu.edu.cn

^#^These authors made equal contributions to this work.

**Supplementary Methods**

**Materials**

Maleimide-PEG-Hydroxy (MAL-PEG-OH Mn 5000), N-Hydroxysuccinimide (NHS), Triethylamine (TEA), N-Methylpyrrolidone (NMP) and N,N-Dimethylformamide (DMF), Methoxy poly(ethylene glycol) (mPEG, average Mn 2000), caprolactone, Sn(Oct)_2_, 11-mercaptoundecanoic acid, 2-hydroxyethyl disulfide, glucosamine hydrochloride, N,N-Diisopropylethylamine (DIPEA) were purchased from Sigma-Aldrich (USA). ARV-825 was purchased from MedChemExpress (USA). Cell Counting Kit-8 (CCK8) was obtained from Dongren Chemical Technology (Japan). Annexin V-FITC/PI apoptosis detection kit was purchased from BD Bioscience. Crystal violet staining solution and cells cycle analysis kit were obtained from meilunbio (China). D-fluorescein potassium was purchased from AAT Bioquest (USA). The Notch1 gene knockout experiment primer sequences: Notch1, 5'-GCTTCCTTCTACTGCGAATGT-3'; NOTCH1, 5'-CCGGGACATCACGGATCATAT-3', were synthesized by Tsingke Biotech Company (China).

**Cell lines and animals**

Murine cerebral cortex endothelial cell bEnd.3, Murine glioma cell GL261, human glioma cell U87, LN229 and U251 cells were bought from American Type Culture Collection (ATCC, USA). GL261-Luc cells and LN229-Luc cells expressing luciferase were constructed by our laboratory. All cells were cultured in Dulbecco's modified Eagle medium (DMEM, Hyclone, USA) containing 10% fetal bovine serum (FBS, Gibco, USA) and 1% streptomycin and penicillin (Hyclone, USA) at 37℃ in 5% CO_2_.

Female C57BL/6 mice and female Balb/c nude mice (6 weeks old) were obtained from Beijing Huafukang Biotechnology (China). All mice were raised under standard condition and animal experiments were performed according to the guidelines approved by the Animal Experimental Ethics Committee of State Key laboratory biotherapy, Sichuan University (Chengdu, China).

**Molecular dynamics simulation**

Molecular dynamics simulation of the interaction between Glu-PEG-PCL, PEG-SS-PCL, ARV-825, and TMZ was performed by NAMD software. Briefly, Glu-PEG-PCL, PEG-SS-PCL, ARV-825, and TMZ were randomly placed within 10 angstrom (0.1 nm) apart from each other, followed by implementing molecular dynamics simulation to calculate and simulate the phase transformation process of the four molecules approaching each other and being embedded in each other in normal physiological environment (pH 7.4, GSH 0.1 mM). The dynamic simulation lasted for 20 ns and was recorded by snapshots every 4 ns. Subsequently, molecular dynamics simulation was performed to investigate the GSH-responsive process of T+A@Glu-NPs in tumor-mimicking high GSH environment (GSH 10 mM). The dynamic simulation also lasted for 20 ns and was recorded by snapshots every 4 ns.

**Preparation of Glu-PEG-PCL and PEG-SS-PCL**

mPEG-COOH (400 mg) was dissolved in N, N-dimethylformamide (DMF) (10 mL), followed by the addition of NHS (46 mg) and EDC (76.4 mg), activated under ice water bath for 1 hour. Subsequently, 2-hydroxyethyl disulfide (61.7 mg) was added, and the mixture was stirred continuously for 24 hours. After dialysis and freeze-drying, PEG-SS-OH was obtained. The structure of PEG-SS-OH was characterized by ^1^H nuclear magnetic resonance（^1^H NMR）. Further, PEG-SS-OH (427 mg), caprolactone (684 mg), and catalytic amount of Sn(Oct)_2_ were reacted in anhydrous toluene in an argon atmosphere argon, and the reaction was carried out at 110℃ for 24 hours. The reaction product was precipitated by dropping into ice-cold petroleum ether, re-dissolved in a small amount of DMF, and after dialysis, PEG-SS-PCL was obtained via freeze drying. The structure was characterized by ^1^H NMR (AVANCE NEO, Bruker, Germany) and infrared spectroscopy (invenior，Bruker，Germany).

The mixture of EDC (1.05 g) and NHS (0.64 g) was added to the DMSO solution of 11-mercaptoundecanoic acid (0.53 g) at room temperature under aegon atmosphere. After stirring for 12 h, glucosamine hydrochloride (1.08 g) and DIPEA (2.5 mL) were added. The mixture was stirred for an additional 12 h, and then poured into cold water. The precipitation was collected, washed with NaOH solution and pure water, finally dried to obtain Glu-SH. The structure was characterized by ^1^H NMR and FT-IR.

MAL-PEG-OH (500 mg), caprolactone (997.5 mg) and catalytic amount of Sn(Oct)_2_ were weighed in a flask. Afterwards, vacuum, drying and air exchange, anhydrous toluene was added to the reaction system to dissolve, and the reaction was reacted for 24 h at 110 °C under argon protection. After the reaction, the solution was sequentially precipitated, dialysed and freeze-dried to obtain MAL-PEG-PCL. The structure of MAL-PEG-PCL was characterized by ^1^H NMR, Subsequently, dried MAL-PEG-PCL (500 mg) and Glu-SH (56.8 mg) were dissolved in DMSO, stirred overnight at room temperature, and dialysed and freeze-dried to obtain actively targeted Glu-PEG-PCL. The structure was characterized by ^1^H NMR and FT-IR.

**The synthesis process of Ce6, Ce6@NPs, and Ce6@Glu-NPs**

For free Ce6 solution, a certain volume of DMSO and Tween 80 was used to ultrasonically dissolve Ce6, followed by the addition of 5% glucose solution to dilute to a Ce6 concentration of 1 mg/mL. For Ce6@NPs, 4 mg of Ce6, 30 mg of PEG-SS-PCL and 10mg of PEG-PCL were dissolved in 500 μL DMSO. The solution was then quickly added to ten times the volume of water and ultrasonicated for an additional 10-15 minutes. The organic phase and free drug were removed by ultrafiltration centrifugation to obtain Ce6@NPs. For Ce6@Glu-NPs, 4 mg of Ce6, 30 mg of PEG-S-S-PCL, and 10 mg of Glu-PEG-PCL were dissolved in 500 μL DMSO. The preparation methods are the same as Ce6@NPs. Both Ce6@NPs and Ce6@Glu-NPs calculated the concentration by measuring ultraviolet absorption.

**In vitro BBB model**

When the cerebral microvascular endothelial cell line bEND.3 is in good growth condition, it is first thoroughly digested with trypsin. Subsequently, the cells are plated at a density of 8×10^4^ to 16×10^4^ per 0.5 mL into the culture chambers of a 12-well plate. Add 1.5 mL of Dulbecco's Modified Eagle Medium (DMEM) containing 20% Fetal Bovine Serum (FBS) to the lower compartment of the chamber. After plating, place the plate in a cell culture incubator and change the medium every three days. Approximately seven days later, the transendothelial electrical resistance (TEER) measurement technique can be initiated to determine the resistance value of the cell layer. A resistance value exceeding 200Ω·cm² indicates that a dense monolayer has been formed, signifying the successful construction of the in vitro blood-brain barrier (BBB) model. After successful construction, Ce6@NPs and Ce6@Glu-NPs nanoparticles are prepared using DMEM medium without phenol red and added to the upper chamber. The culture continues in the cell culture incubator. At the time points of 1, 2, 4, 6, 12, and 24 hours, 100 μL of the medium from the lower compartment is aspirated and replaced with the same volume of DMEM without phenol red. Fluorescence intensity is measured using a microplate reader, and the corresponding fluorescence ratios are calculated to assess the transport efficiency of the nanoparticles across the cell layer.

**Immunohistochemistry, TUNEL and H&E staining assay**

Tumors and major organ harvested after treatment were fixed with 4% paraformaldehyde, embedded in paraffin and sliced. In immunohistochemistry assay, tumor slices went through the steps of baking, dewaxing, antigen repair, removal of endogenous catalase, blocking, incubation of primary antibody (Ki67 polyclonal IgG, Servicebio, China), incubation of secondary antibody, DAB color development, hematoxylin re-staining and sealing. The staining results of Ki67 were observed and photographed through an upright microscope (Olympus, Japan) for evaluation of tumor cells proliferation and angiogenesis, respectively. TUNEL assay was perform according to the TUNEL apoptosis detection kit (BD Bioscience, USA) to analyze the apoptosis of tumor cells. For sections requiring H&E staining, dewaxing, staining (hematoxylin staining the nucleus, eosin staining the cytoplasm), dehydration and sealing were carried out in turn. The organ tissues were observed and photographed with the microscope, and the tumor tissues in situ were observed with a pathological scanner (3DHISTECH, Hungary).

**Blood biochemical analysis**

The blood of mice in each group was placed at 4℃ for one night and then was centrifuged at 4℃ (2000 rpm, 15 min), the upper serum was collected to detect some momentous blood biochemical indexes with the blood biochemical instrument (Roche, Switzerland).

**Western blot analysis**

Proteins were cleaved with RIPA lysis buffer (Beyotime Biotechnology, China) containing 1% protease inhibitor cocktail and quantified according to manufacturer’s instruction of BCA protein quantitative kit (Beyotime Biotechnology, China). Thereafter, proteins were performed SDS/PAGE gels followed by transferring proteins to PVDF blotting membrane, blocking with TBST containing 5% BSA or skimmed milk, incubating primary antibodies at 4℃ overnight and incubating second antibodies at room temperature for 1-2 hours. The bands of proteins were developed with an ECL developer (Beijing Juhemei Biotechnology, China) and visualized by a chemiluminescence imaging system (Clinx Science Instruments, China). The primary antibodies and second antibodies in this study were displayed in Supplementary Table S2.

**RT-qPCR**

The RNA of BMDMs was extracted with UNlQ-10 column total RNA purification Kit (Sangon Biotech, China) and cDNA was obtained according to the instruction of HiScript III SuperMix for qPCR (+gDNA wiper) (Vazyme, China). The qPCR assay was implemented by a LightCycler®480 real-time fluorescent quantitative PCR instrument (Roche, Switzerland). Primers used were listed in supplementary table S1. The relative content of objective genes was calculated by the 2^-△△CT^ method with GAPDH as the internal gene.

**Supplementary Tables**

**Table S1** Information of antibodies used in western blot analysis.

| Antibodies | Company | Catalog number |
| --- | --- | --- |
| BRD4 | Abcam | ab128874 |
| Notch1 | Cell Signaling Technology (CST) | #3608 |
| Cyclin D1 | CST | #55506 |
| CDK4 | CST | #12790 |
| Caspase-3 | CST | #9662 |
| Cleaved Caspase-3 | CST | #9661 |
| PARP/Cleaved PARP | CST | #9542 |
| Stat3 | CST | #12640 |
| Phospho-Stat3 | CST | #9145 |
| GAPDH | absin | abs137959 |
| Goat anti-mouse IgG-HRP | ZSGB-BIO | ZB-2305 |
| Goat anti-rabbit IgG-HRP | ZSGB-BIO | ZB-2301 |

**Table S2** Sequences of primers used in RT-qPCR assay

| Gene |  | Sequence |
| --- | --- | --- |
| Notch1 | Forward | 5'-GATGGCCTCAATGGGTACAAG-3' |
|  | Reverse | 5'-TCGTTGTTGTTGATGTCACAGT-3' |
| Maml2 | Forward | 5'-TTTCCTTGGCTAACTCTGCAC-3' |
|  | Reverse | 5'-CCCTGTTTGCTCCTGATACTG-3' |
| Ccn3 | Forward | 5'-AGTGCCCCAGTATATCACCGA-3' |
|  | Reverse | 5'-TGCGGTCACAGTAGAGACCA-3' |
| Ptp4a3 | Forward | 5'-CATCACTGTTGTGGACTGGC-3' |
|  | Reverse | 5'-TGGATGGCGTCCTCGTACTT-3' |
| Fat4 | Forward | 5'-CAGTGGTGATCCAGGTACGG-3' |
|  | Reverse | 5'-TCATGCGCTGTCACGGAAATA-3' |
| Cdh6 | Forward | 5'-GAGAACACAGGCGACATACAG-3' |
|  | Reverse | 5'-GACCACAAATGTGCCAACATC-3' |
| NOTCH1 | Forward | 5'-CGCTGACGGAGTACAAGTG-3' |
|  | Reverse | 5'-GTAGGAGCCGACCTCGTTG-3' |
| Gapdh | Forward | 5'-ACCCTTAAGAGGGATGCTGC-3' |
|  | Reverse | 5'-CCCAATACGGCCAAATCCGT-3' |
| GAPDH | Forward | 5'-GAAAGCCTGCCGGTGACTAA-3' |
|  | Reverse | 5'-GCCCAATACGACCAAATCAGAG-3' |

**Table S3** Sequences of primers used in ChIP assay.

| Primer |  | Sequence |
| --- | --- | --- |
| Primer1 | Forward | 5'-TCTATGCTGCCCATCATACCTGC-3' |
|  | Reverse | 5'-CAGCCTCAGATGTTCTGTTGCTGA-3' |
| Primer2 | Forward | 5'-GGCTGAGTGAGTATTTAGCAGGAAGAG-3' |
|  | Reverse | 5'-CCAGCCAACCAACAGAAACAACAG-3' |
| Primer3 | Forward | 5'-TTACCCTCCAAGCCCTTTTCCTG -3' |
|  | Reverse | 5'-CTCAAGTTCTTGGCAAGTGGGTGT-3' |
| Primer4 | Forward | 5'-ATTTTTCTGGCTCAGCTGTCCCAG-3' |
|  | Reverse | 5'-GTCTAGCAATGAGTATGGCTGCACAG-3' |
| Primer5 | Forward | 5'-AGACAGTGGAAGAGATACAGACACC-3' |
|  | Reverse | 5'-GAAGGCCTTATCCTTCTGGAGTCT -3' |
| Primer6 | Forward | 5'-CTTTGTATCCAAGTCAGGCTGCCT-3' |
|  | Reverse | 5'-CTGCAAGTGCCTCTTCTGAGATGG-3' |
| Primer7 | Forward | 5'-TCAGGCGTTCCTTCTATGGAACTG-3' |
|  | Reverse | 5'-GGTCAGTACCCGAGAGAAAAAGCG-3' |
| Primer8 | Forward | 5'-CTTGAGGCCAGTTTCTGAGAGGAG-3' |
|  | Reverse | 5'-CATCTCCCTTGACACACCTGTTCC-3' |
| Primer9 | Forward | 5'-GTCCTAGGACAATATAATCTGGGGCC-3' |
|  | Reverse | 5'-CCAAGATGCCTTCCTTATCCCTTTG-3' |
| Primer10 | Forward | 5'-TGCTAAGCAGCTTAGCACAGGATG-3' |
|  | Reverse | 5'-GTGCAGAGTTCCAGTACAATGACTGC-3' |
| Primer11 | Forward | 5'-TACTGGAACTCTGCACTTTCCCAG-3' |
|  | Reverse | 5'-GAACCCGAGTCTAGACGTTCCA-3' |
| Primer12 | Forward | 5'-AATCGAGAGCGCAATCTGTCCAG-3' |
|  | Reverse | 5'-CTCTTAGCCATGACTTCCGGAACG-3' |
| Primer13 | Forward | 5'-TCAATCCTCCTGATGCCTATAGCC-3' |
|  | Reverse | 5'-GAATCCTGGGGCAGGAGTTTAAATG-3' |
| Primer14 | Forward | 5'-TCCTAGACCTAACTCCAGGATGGA-3' |
|  | Reverse | 5'-GAAGGATCAGGCTTTGTGTGTAGC-3' |
| Primer15 | Forward | 5'-GCCAGCTCAAACTTTTGGTCTCT-3' |
|  | Reverse | 5'-GAGCCAGGATCTAAGGATCTTATGGT -3' |

**Supplementary Figures**


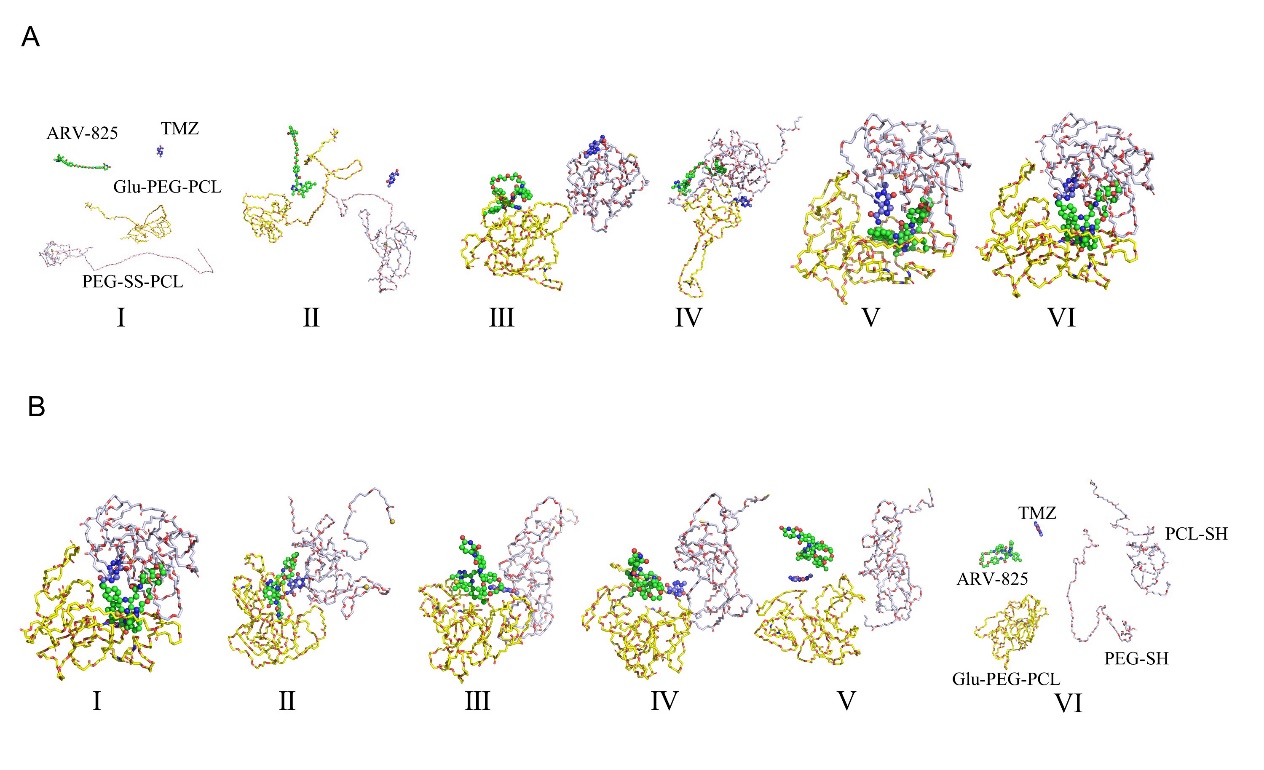


**Figure S1.** Molecular dynamics simulation analysis (A) the self-assembly of TMZ, ARV-825 and Glu-PEG-PCL/PEG-SS-PCL in the normal physiology environment (PH 7.4, GSH 0.1 mM). (B) the GSH responsive and drug release process of T+A@Glu-NPs in high GSH environment (GSH 10 mM). Conformations (I), (II), (III), (IV), (V), and (VI) represented the snapshots of the interaction between TMZ, ARV-825 and Glu-PEG-PCL/PEG-SS-PCL at 0, 4, 8, 12, 16 and 20 ns, respectively.


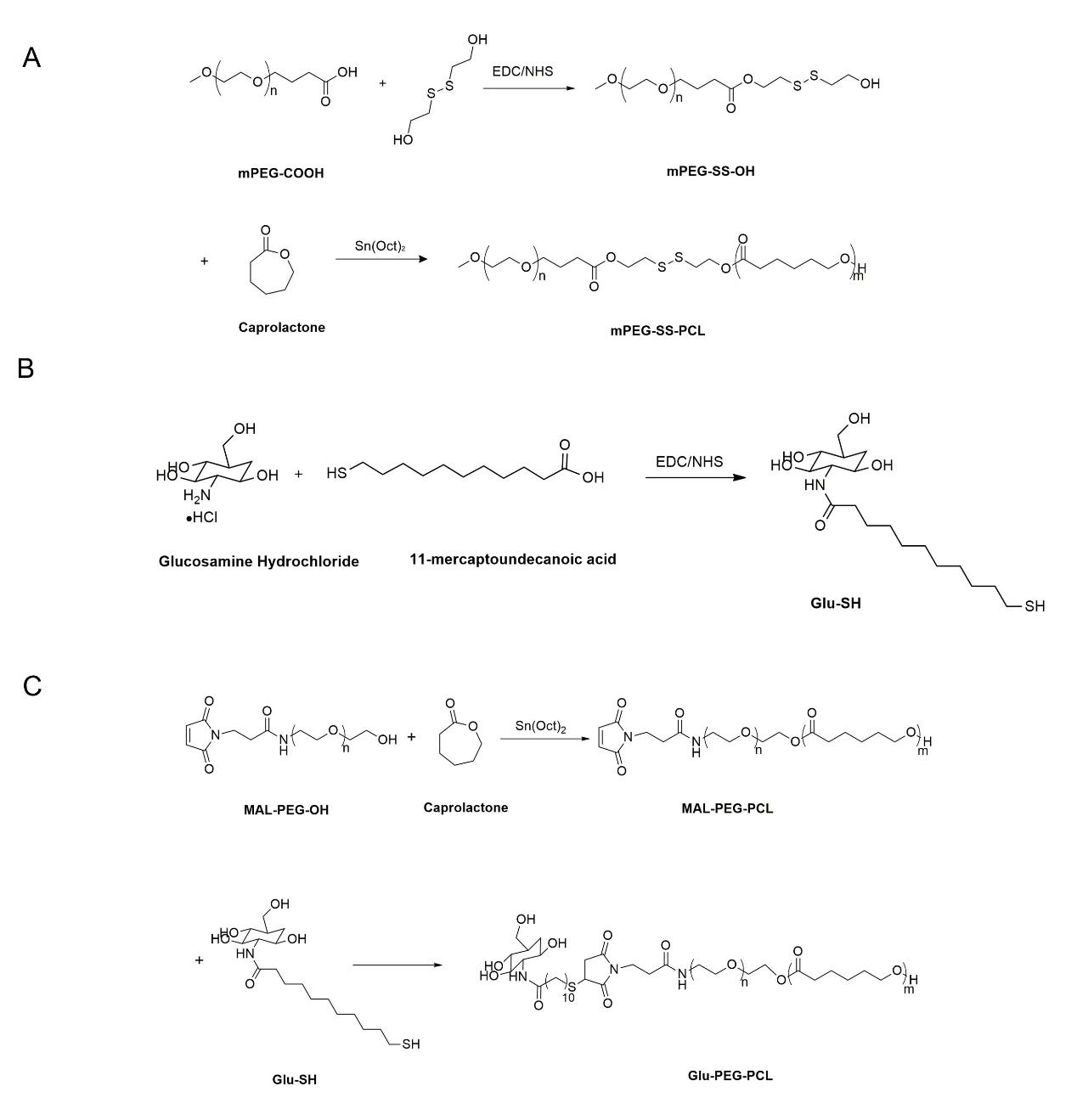


**Figure S2.** The synthetic routes of PEG-SS-PCL and Glu-PEG-PCL (A) the synthesis of PEG-SS-PCL. (B) and (C) the synthesis of Glu-PEG-PCL.


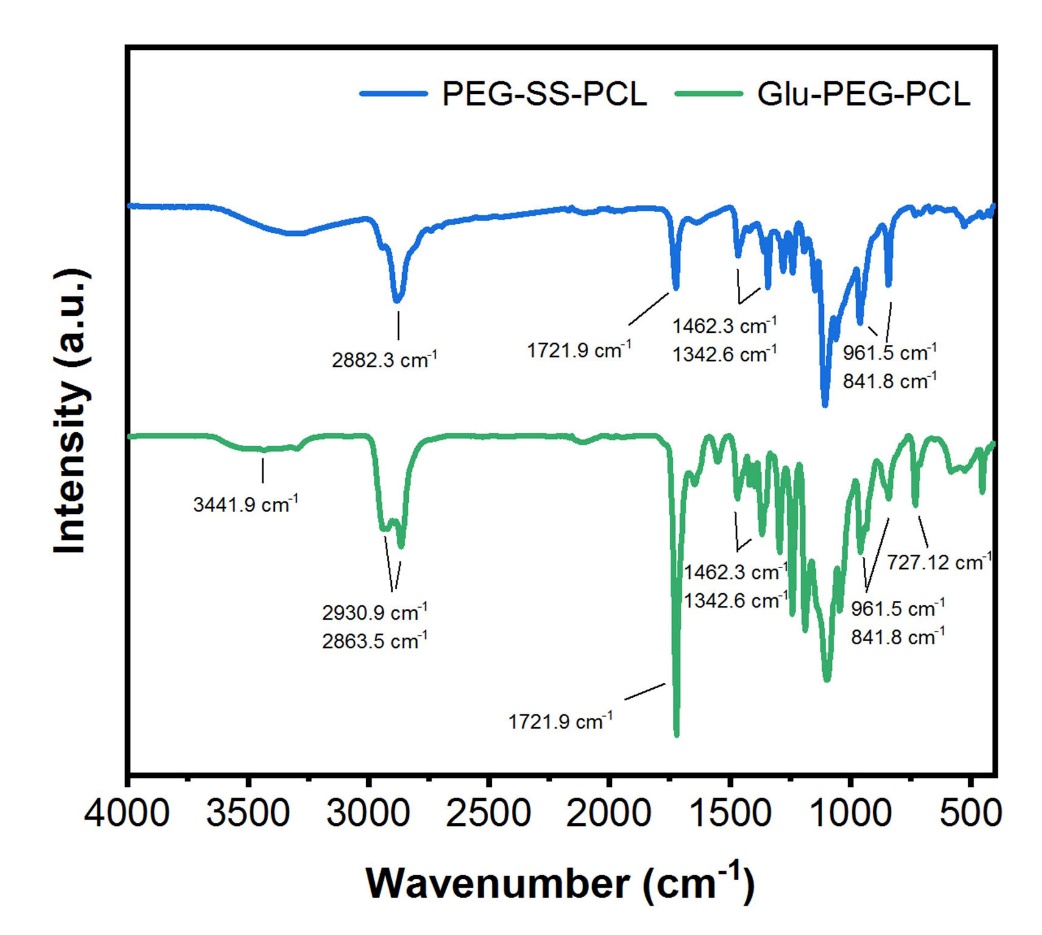


**Figure S3.** The infrared spectroscopy analysis of PEG-SS-PCL and Glu-PEG-PCL The blue curve represents PEG-SS-PCL, the green curve represents Glu-PEG-PCL.


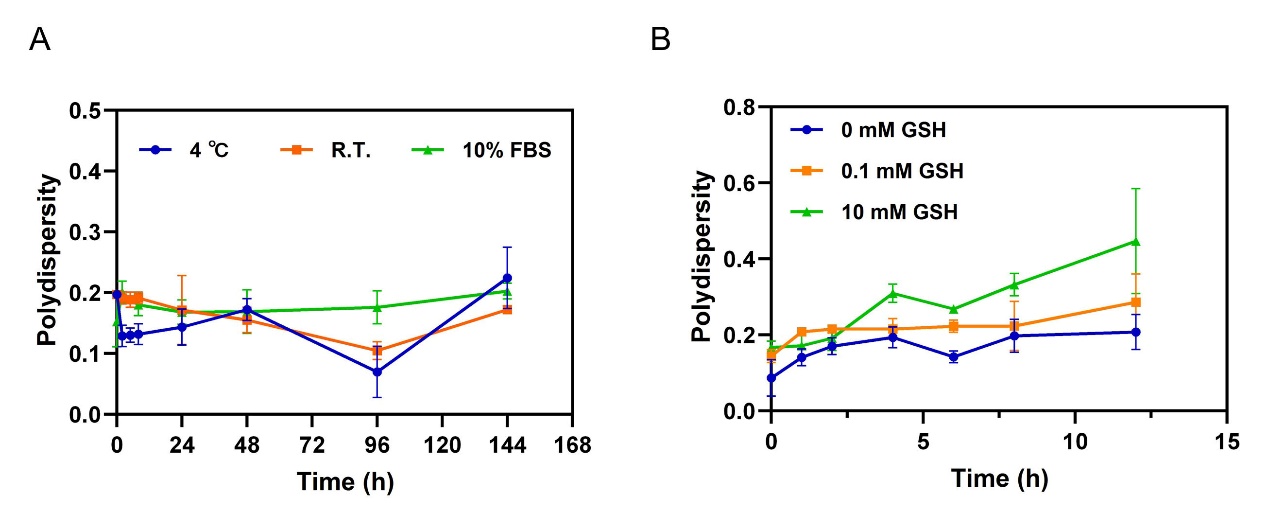


**Figure S4.** The polydispersity of the T+A@Glu-NPs (A) the PDI of the T+A@Glu-NPs in the stablility test. (B) the PDI of the T+A@Glu-NPs in the GSH-responsive test.


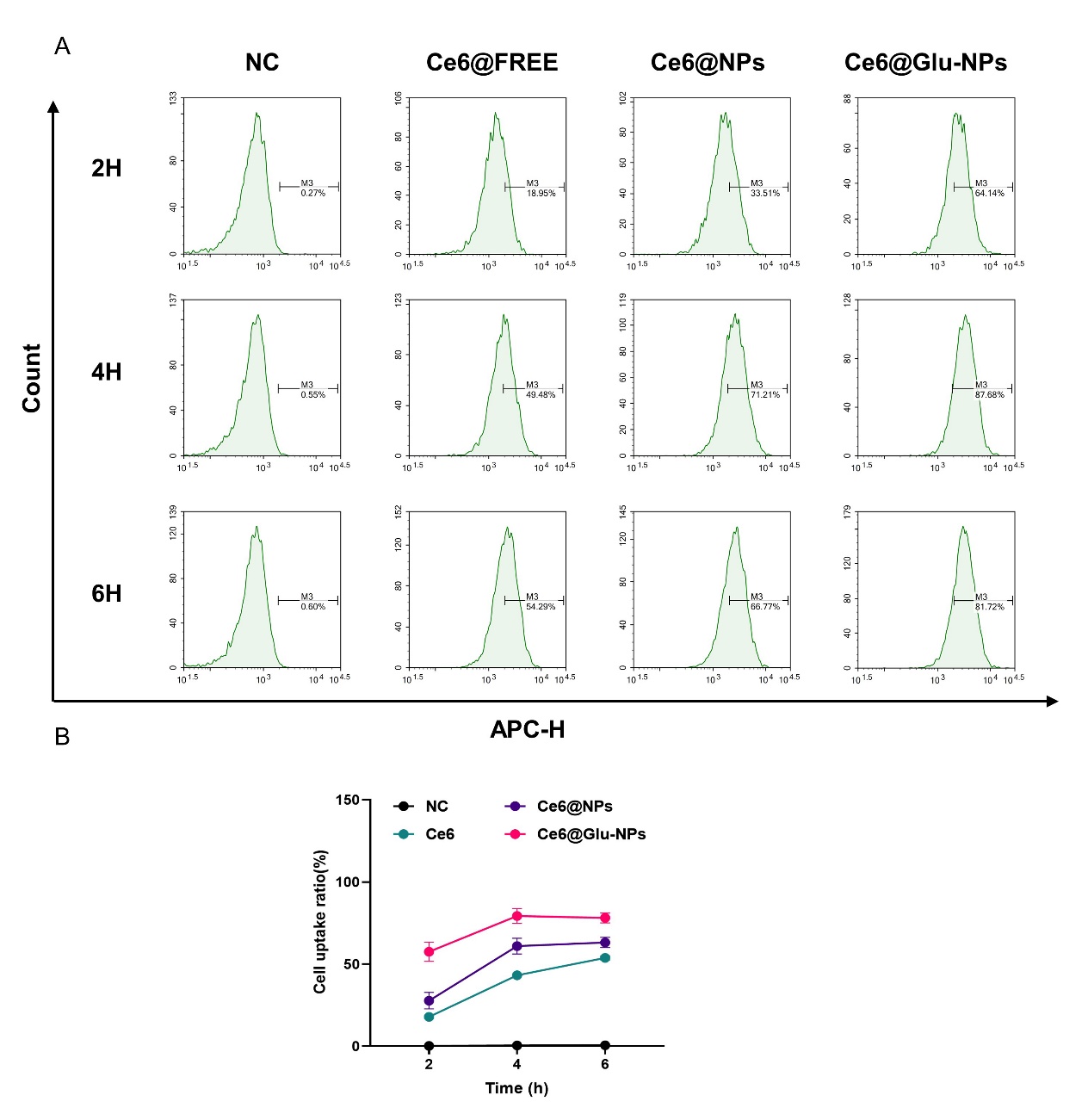


**Figure S5.** The cell uptake assay of GL261 (A) Flow analysis of Ce6 fluorescence counts after incubation with Ce6, Ce6@NPs, Ce6@Glu-NPs for 2 h, 4 h and 6 h. (B) Cellular uptake rate curves for different treatment groups. The untreated group as the control (n=3).


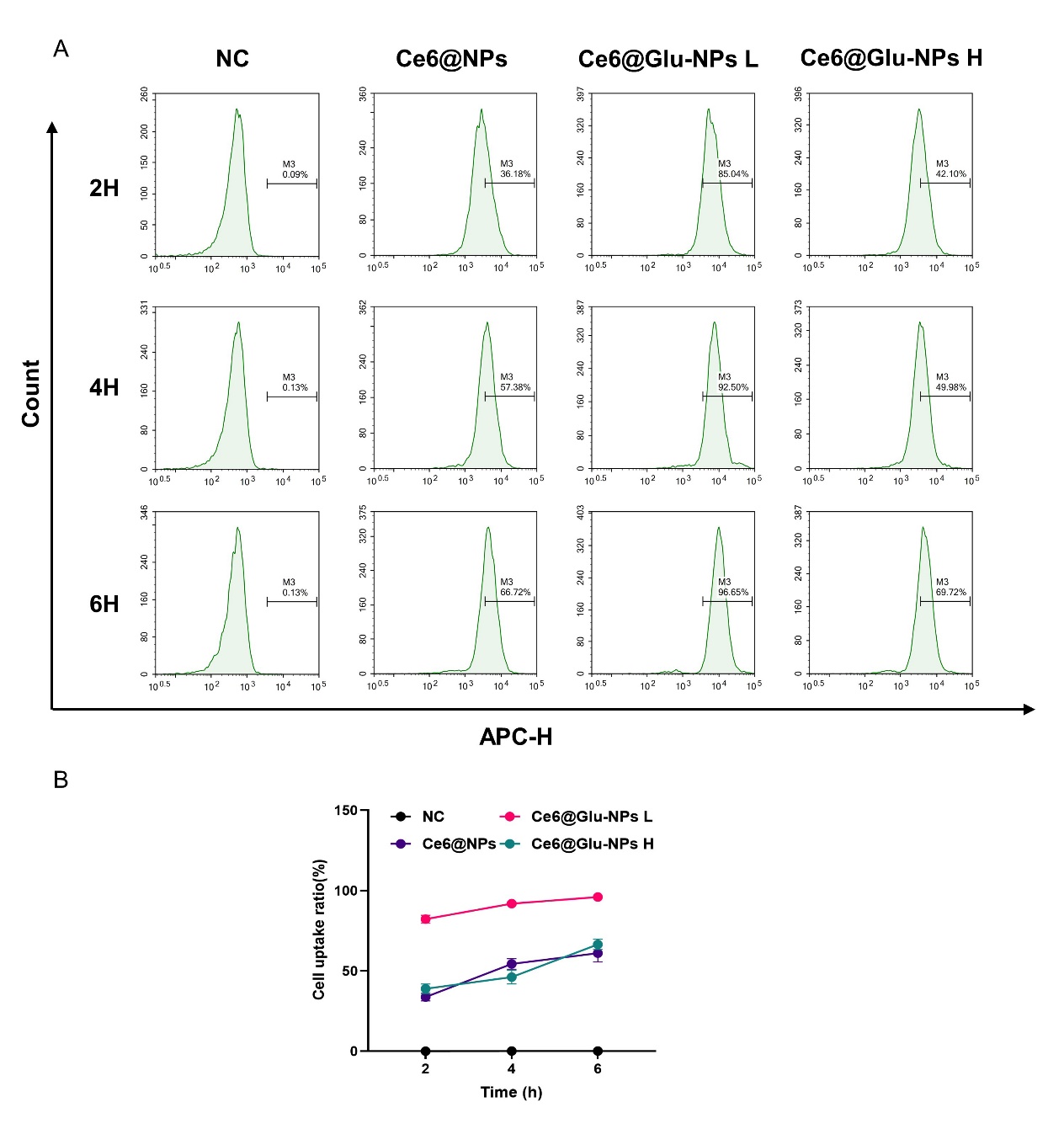


**Figure S6.** The cell uptake assay of GL261 (A) Flow analysis of Ce6 fluorescence counts after incubation with Ce6@NPs, Ce6@Glu-NPs L, Ce6@Glu-NPs H for 2 h, 4 h and 6 h. (B) Cellular uptake rate curves for different treatment groups. The untreated group as the control (n=3).


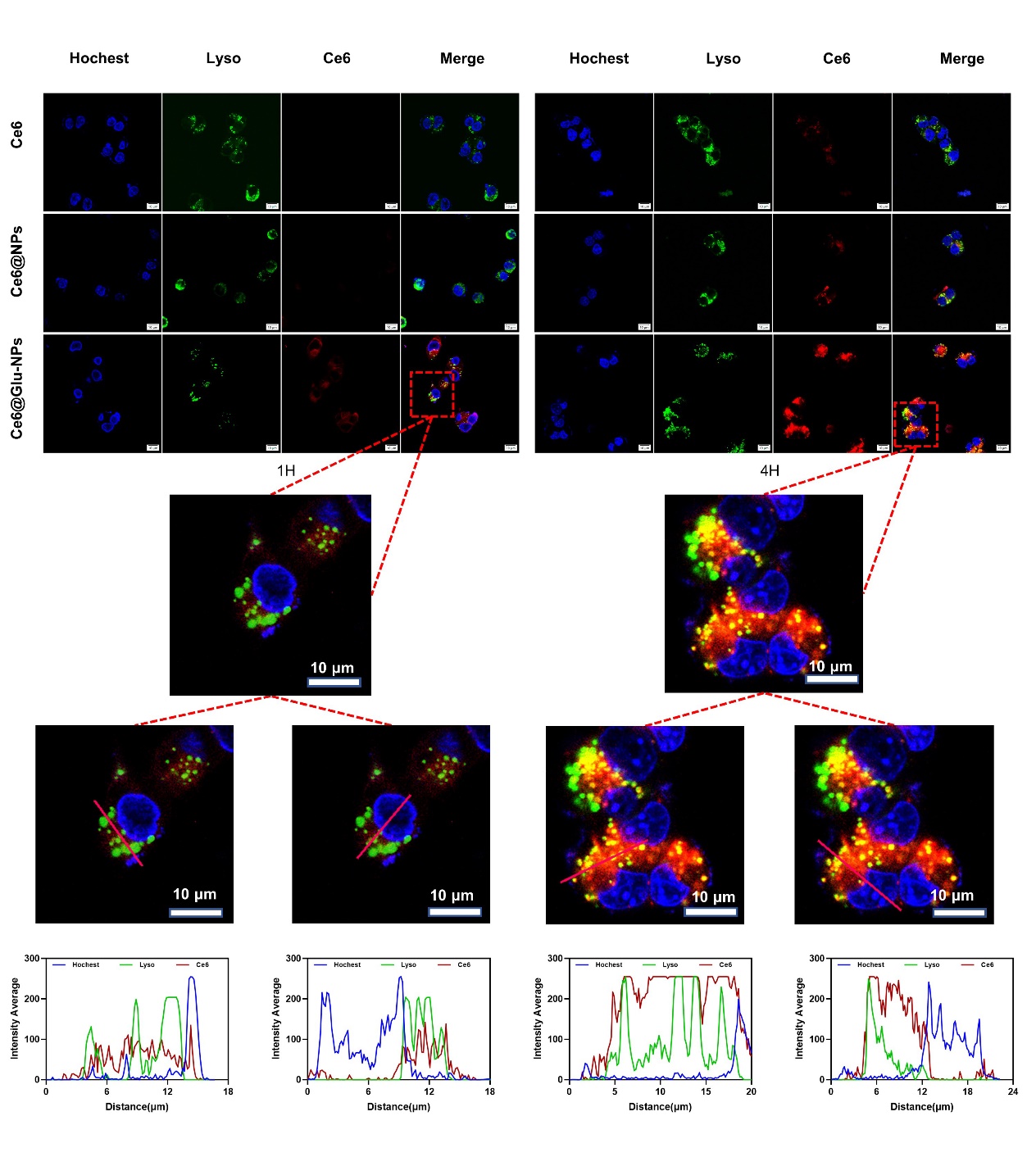


**Figure S7.** The confocal images of GL261. The distribution of Ce6, Hochest33442 and LysoTracker Green DND-26 in GL261 cells was examined using confocal imaging after incubation with Ce6, Ce6@NPs and Ce6@Glu-NPs for 1h and 4h.


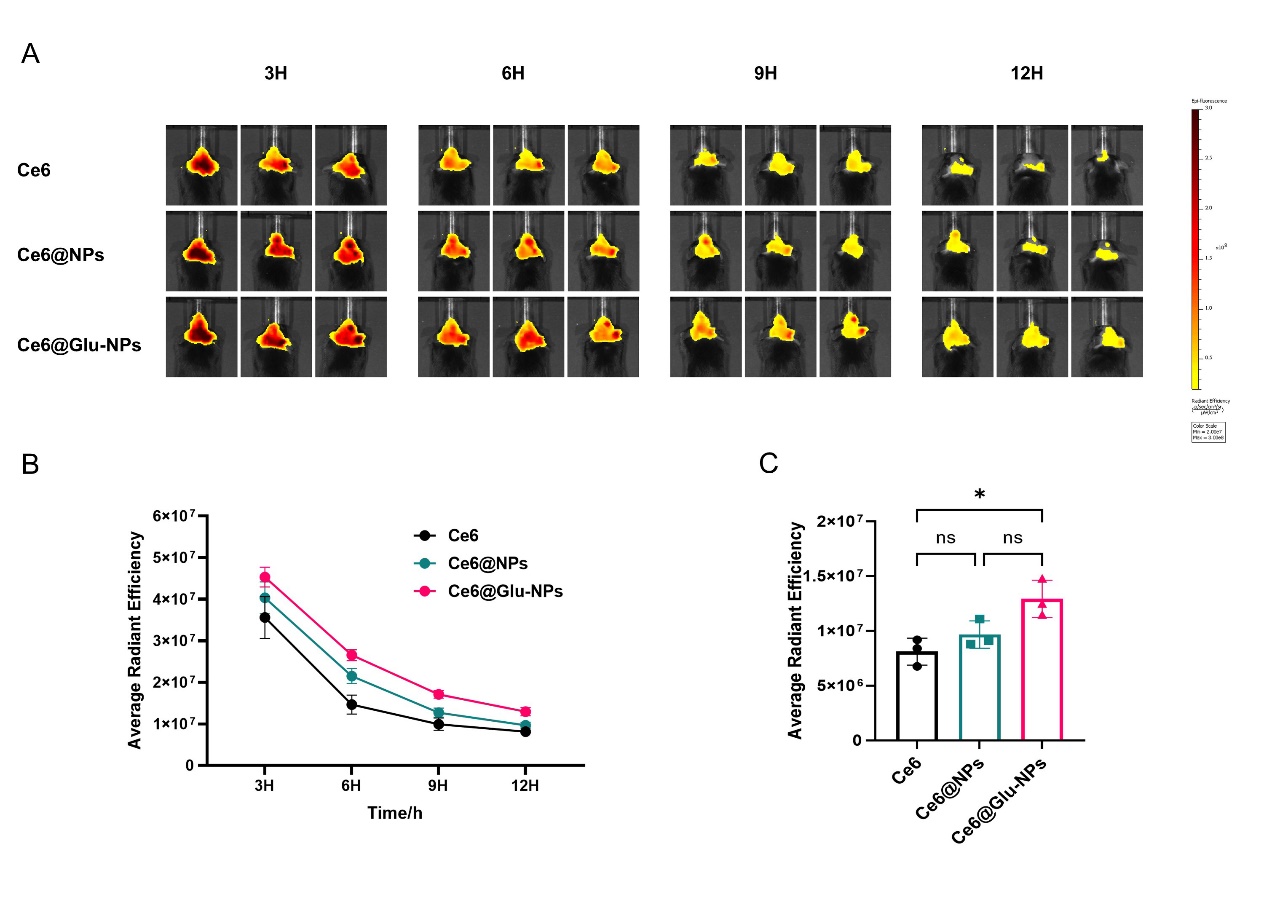


**Figure S8.** In vivo targeting experiments with the GL261 orthotopic glioma model (A) After tail vein injection of Ce6, Ce6@NPs, Ce6@Glu-NPs, the images of the total radiant efficiency of Ce6 in mouse brain. (B) The average radiant efficiency of Ce6 metabolic profile. (C) The average radiant efficiency of Ce6 in mouse brain at 12 h (n=3). Data are presented as mean ± SD. No significant difference is marked with ns. *P < 0.05, **P < 0.01, ***P < 0.001 and ****P < 0.0001.


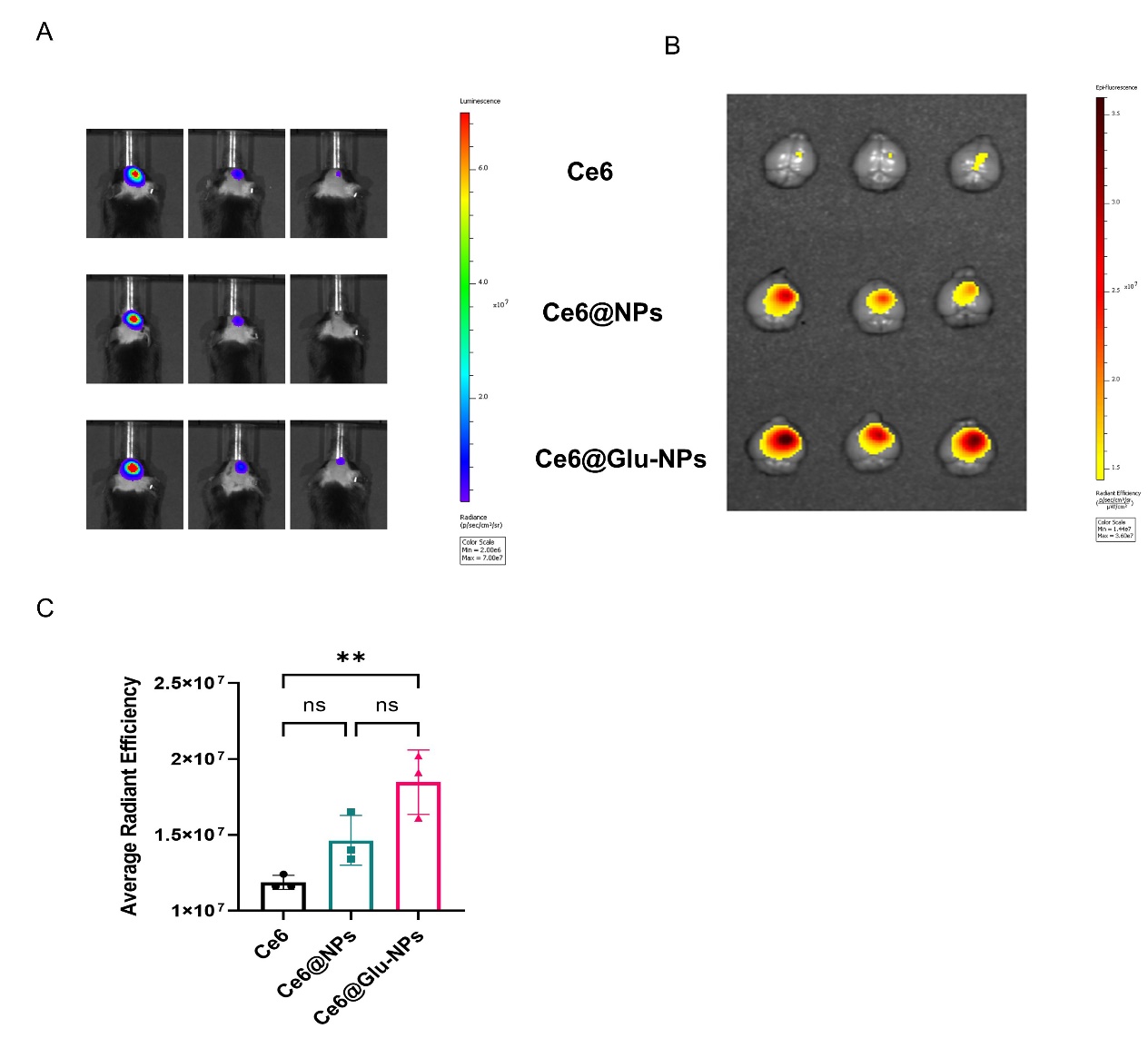


**Figure S9.** In vivo targeting experiments with the GL261 orthotopic glioma model (A) The brain tumor fluorescence intensity images at 0 h. (B) The total radiant efficiency images of Ce6 in mouse brain at 24 h. (C) The average radiant efficiency of Ce6 in mouse brain at 24 h (n=3). Data are presented as mean ± SD. No significant difference is marked with ns. *P < 0.05, **P < 0.01, ***P < 0.001 and ****P < 0.0001.


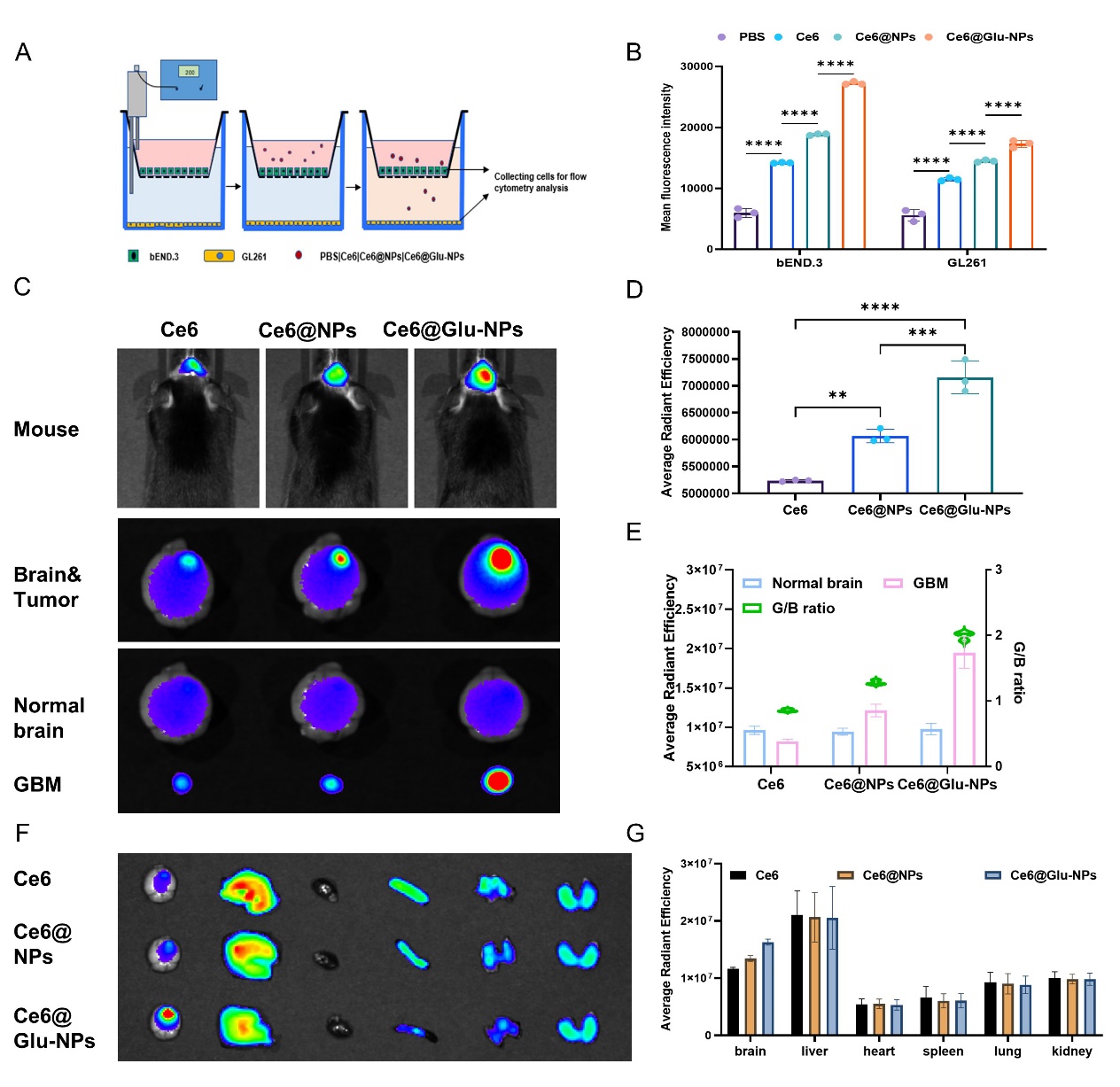


**Figure S10.** Dual-targeting functionality verification (A)Schematic diagram of in vitro BBB model validation of Glu-PEG-PCL's ability to target and penetrate the BBB and target GL261 cells. (B) Flow cytometry analysis of the fluorescence intensity of Glu-PEG-PCL targeting bEND.3 and glioma cells GL261. (C) Fluorescence images of mouse brains after intravenous injection of Ce6, Ce6@NPs, and Ce6@Glu-NPs for 24 hours, ex vivo brain tissue and tumor fluorescence images, and fluorescence images of separated normal brain tissue and GBM. (D) Statistical graph of average fluorescence intensity in mouse brains. (E) Statistical graph of average fluorescence intensity in normal brain tissue and GBM, as well as a statistical graph of their ratio. (F) Ex vivo fluorescence images of brain and important organs after intravenous injection of Ce6, Ce6@NPs, and Ce6@Glu-NPs for 24 hours. (G) Statistical graph of average fluorescence intensity distribution in various organs. Data are presented as mean ± SD. No significant difference is marked with ns. *P < 0.05, **P < 0.01, ***P < 0.001 and ****P < 0.0001.


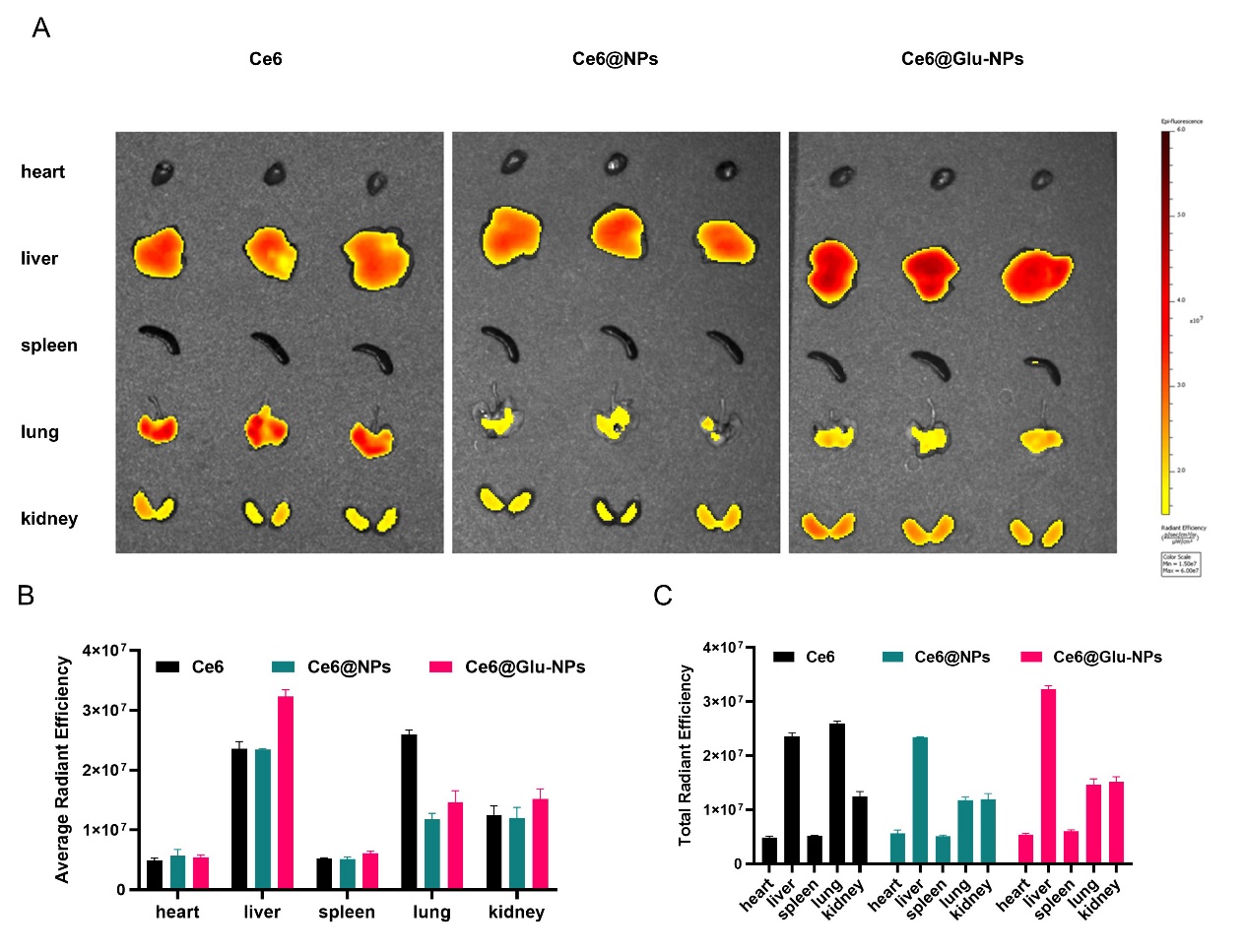


**Figure S11.** In vivo targeting experiments with the GL261 orthotopic glioma model (A) Images of Ce6 distribution in vital organs. (B) and (C) Statistical analysis of Ce6 average radiant efficiency in vital organs (n=3). Data are presented as mean ± SD. No significant difference is marked with ns. *P < 0.05, **P < 0.01, ***P < 0.001 and ****P < 0.0001.


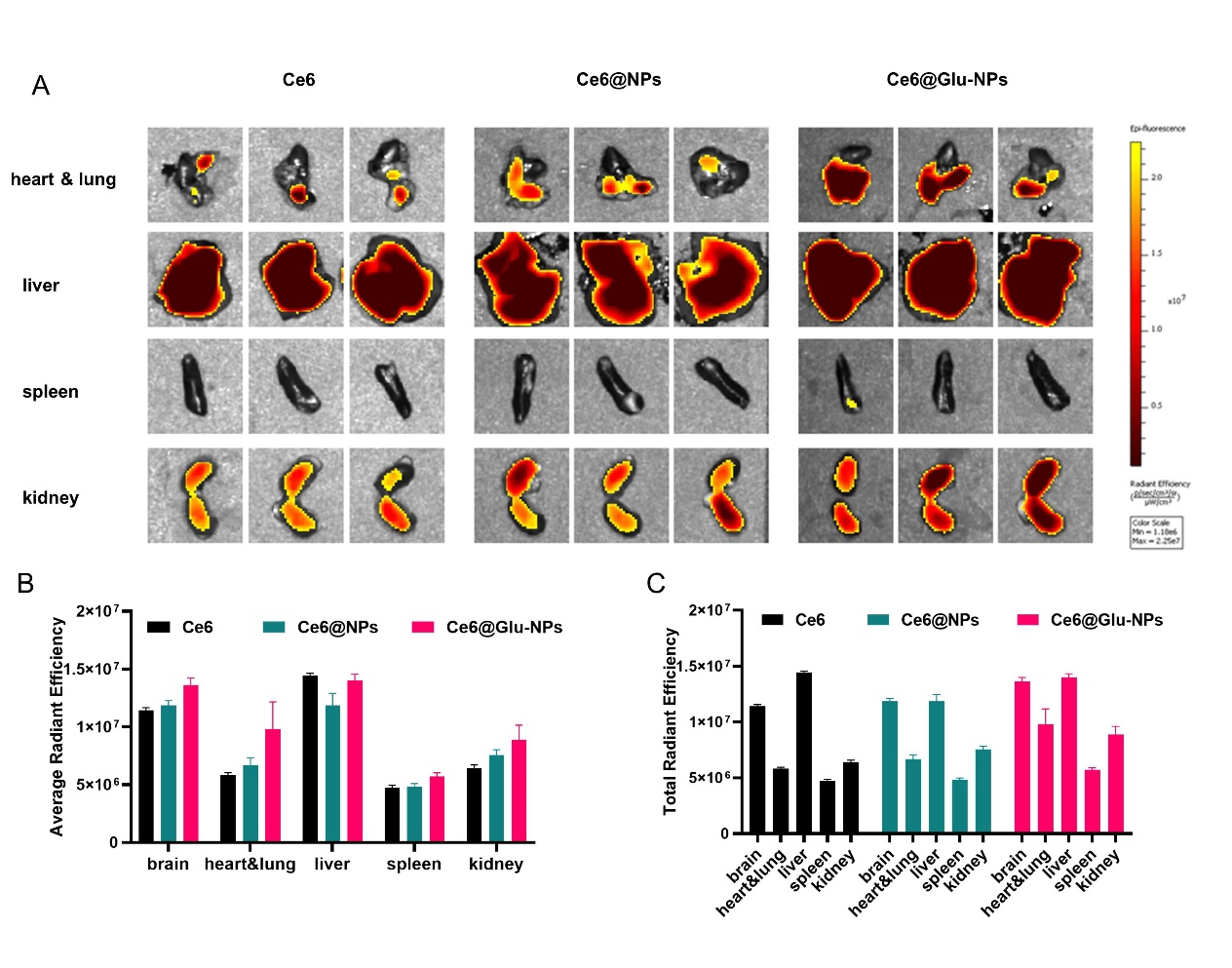


**Figure S12.** In vivo targeting experiments with the LN229 orthotopic glioma model (A) Images of Ce6 distribution in vital organs. (B) and (C) Statistical analysis of Ce6 average radiant efficiency in vital organs (n=3). Data are presented as mean ± SD. No significant difference is marked with ns. *P < 0.05, **P < 0.01, ***P < 0.001 and ****P < 0.0001.


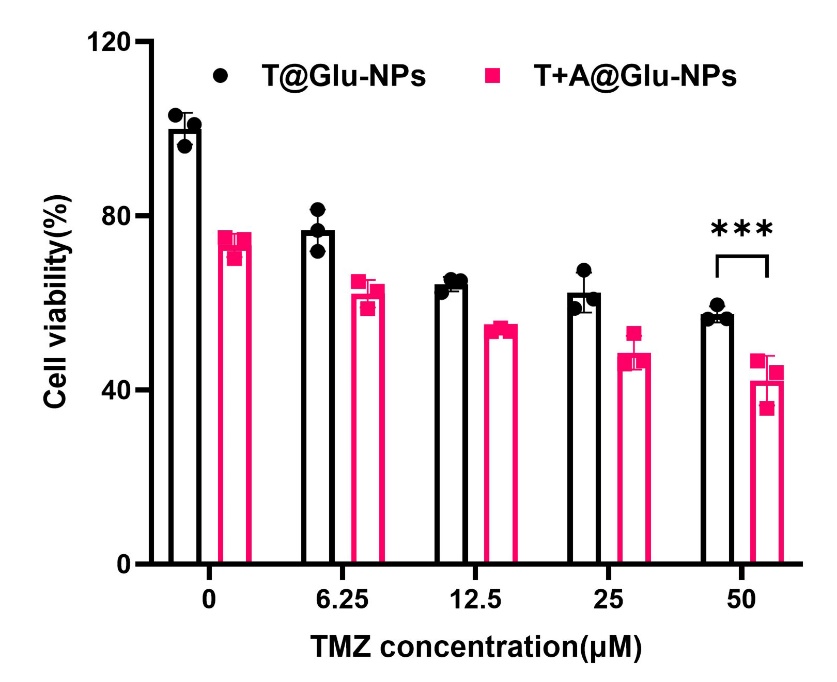


**Figure S13.** Cell viabilities of LN229 cells treated with A@Glu-NPs at 120 ng/mL, T@Glu-NPs and T+A@Glu-NPs at different concentrations for 48 h, the untreated group as a control group (n = 3). Data are presented as mean ± SD. No significant difference is marked with ns. *P < 0.05, **P < 0.01, ***P < 0.001 and ****P < 0.0001.


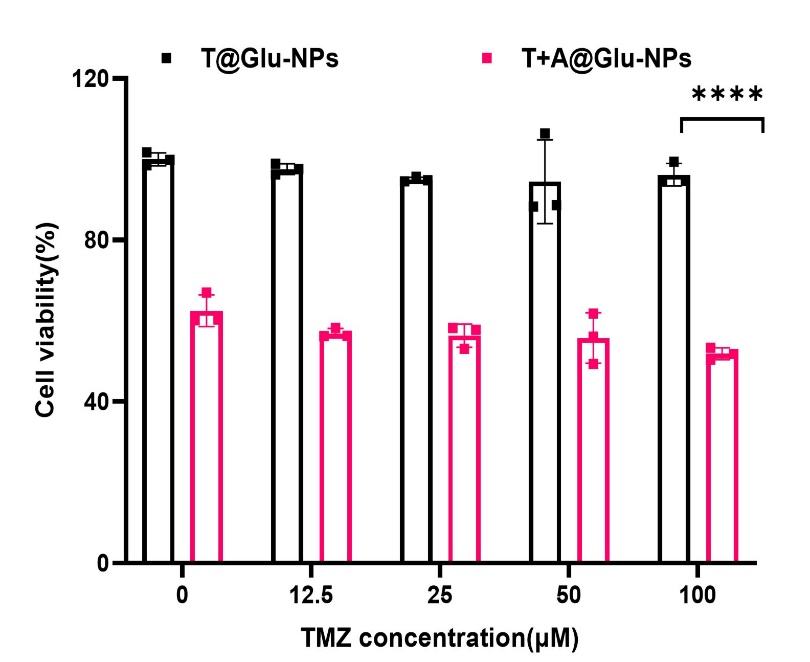


**Figure S14.** Cell viabilities of U87 cells treated with A@Glu-NPs at 50 ng/mL, T@Glu-NPs and T+A@Glu-NPs at different concentrations for 48 h, the untreated group as a control group (n = 3). Data are presented as mean ± SD. No significant difference is marked with ns. *P < 0.05, **P < 0.01, ***P < 0.001 and ****P < 0.0001.


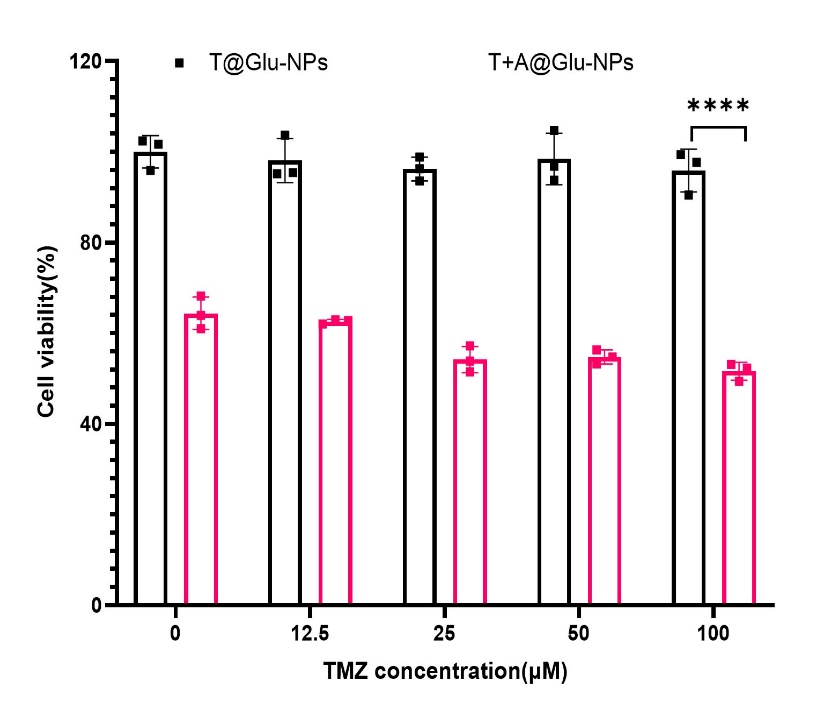


**Figure S15.** Cell viabilities of U251 cells treated with A@Glu-NPs at 50 ng/mL, T@Glu-NPs and T+A@Glu-NPs at different concentrations for 48 h, the untreated group as a control group (n = 3). Data are presented as mean ± SD. No significant difference is marked with ns. *P < 0.05, **P < 0.01, ***P < 0.001 and ****P < 0.0001.


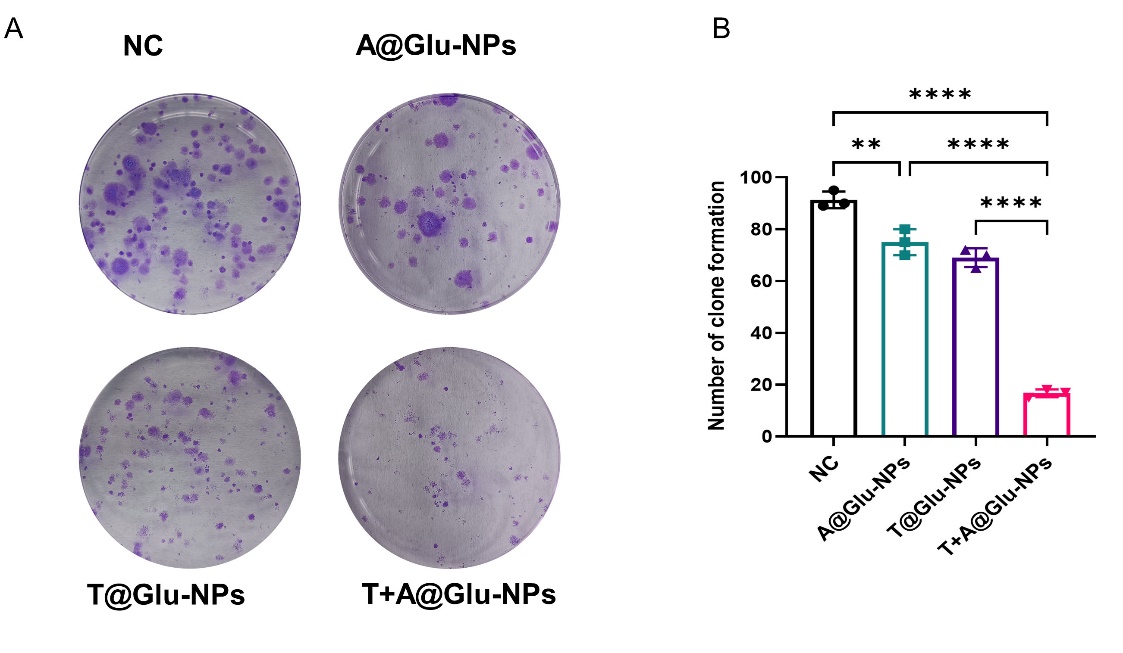


**Figure S16.** The colony formation assay of LN229 cells (A) The colony formation images of LN229 cells after treated with A@Glu-NPs at 25 ng/mL, T@Glu-NPs at 0.78125 μM and T+A@Glu-NPs for two weeks, the untreated group as a control group. (B) Statistical analysis of the clone numbers of LN229 cells (n = 3). Data are presented as mean ± SD. No significant difference is marked with ns. *P < 0.05, **P < 0.01, ***P < 0.001 and ****P < 0.0001.


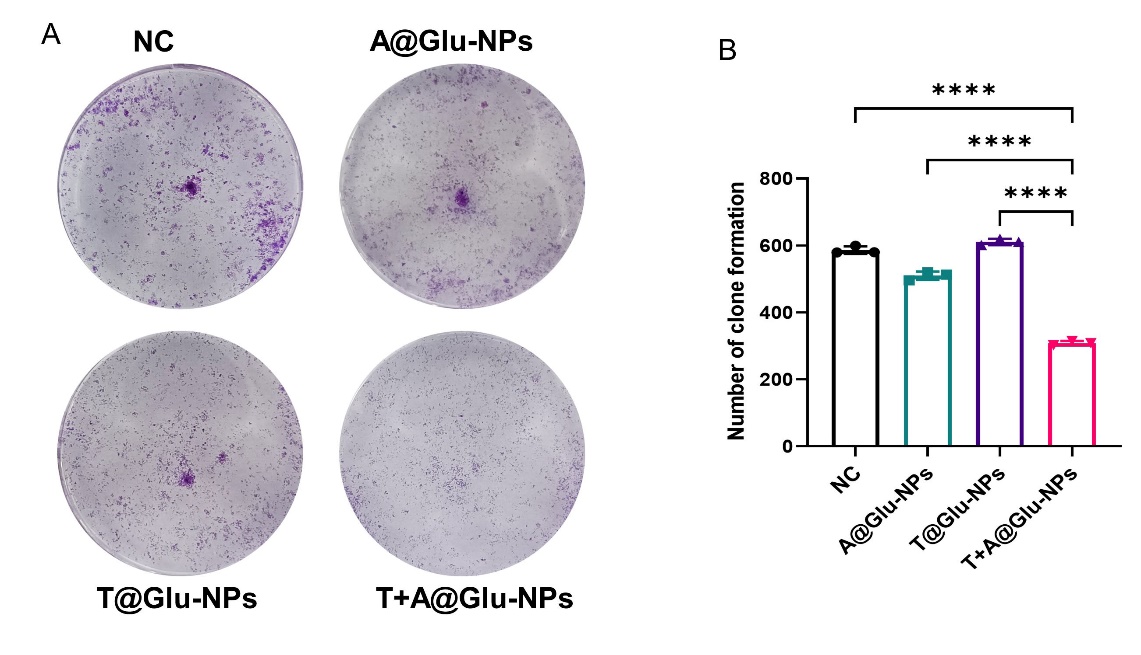


**Figure S17.** The colony formation assay of U251 cells (A) The colony formation images of U251 cells after treated with A@Glu-NPs at 10 ng/mL, T@Glu-NPs at 25 μM and T+A@Glu-NPs for two weeks, the untreated group as a control group. (B) Statistical analysis of the clone numbers of U251 cells (n = 3). Data are presented as mean ± SD. No significant difference is marked with ns. *P < 0.05, **P < 0.01, ***P < 0.001 and ****P < 0.0001.


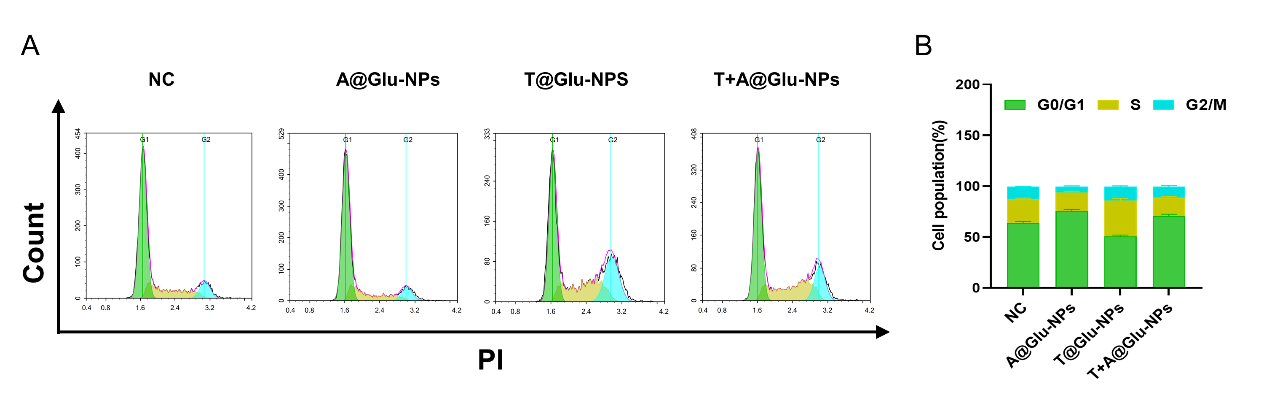


**Figure S18.** Flow cycle analysis of LN229 cells (A) Flow cycle analysis of LN229 cells after treated with A@Glu-NPs at 120 ng/mL, T@Glu-NPs at 50 μM and T+A@Glu-NPs for 48 h, the untreated group as a control group. (B) Statistical analysis of LN229 cells (n = 3). Data are presented as mean ± SD.


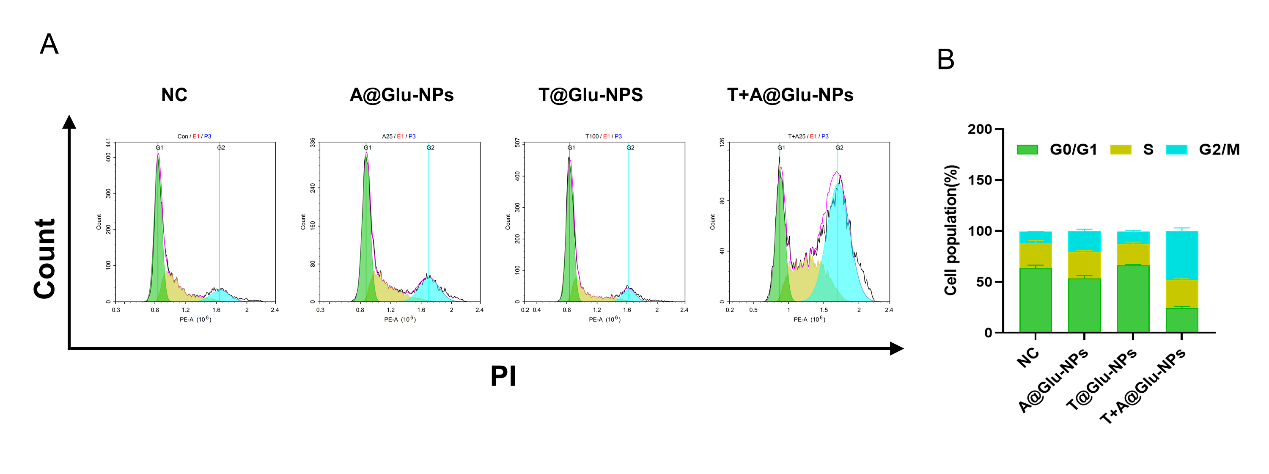


**Figure S19.** Flow cycle analysis of U251 cells (A) Flow cycle analysis of U251 cells after treated with A@Glu-NPs at 25 ng/mL, T@Glu-NPs at 100 μM and T+A@Glu-NPs for 48 h, the untreated group as a control group. (B) Statistical analysis of U251 cells (n = 3). Data are presented as mean ± SD.


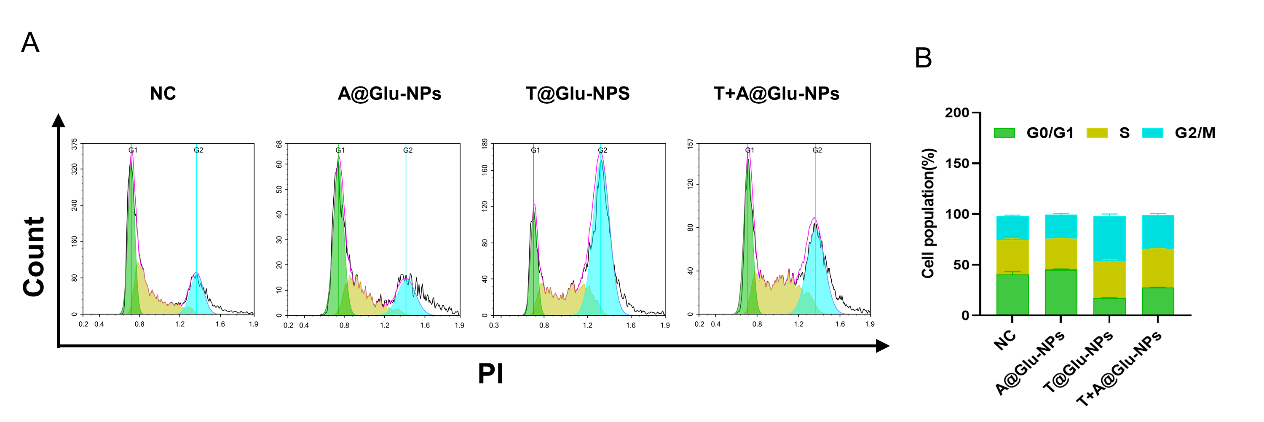


**Figure S20.** Flow cycle analysis of U87 cells (A) Flow cycle analysis of U87 cells after treated with A@Glu-NPs at 50 ng/mL, T@Glu-NPs at 100 μM and T+A@Glu-NPs for 48 h, the untreated group as a control group. (B) Statistical analysis of U87 cells (n = 3). Data are presented as mean ± SD.


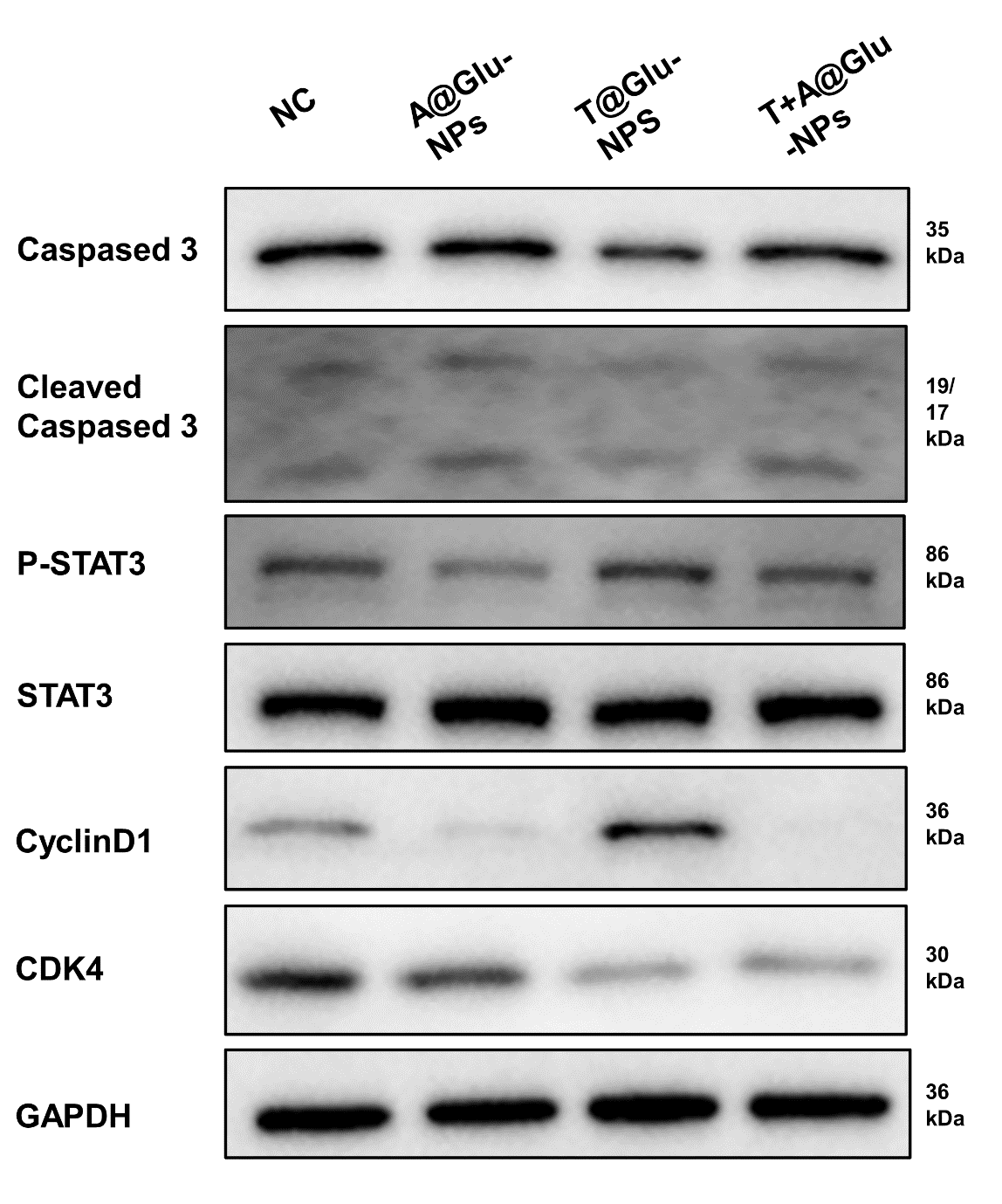


**Figure S21.** Western blot analysis of LN229 cells. LN229 cells were treated with A@Glu-NPs at 200 ng/mL, T@Glu-NPs at 100μM and T+A@Glu-NPs for 48 h, the untreated group as a control group (n = 3).


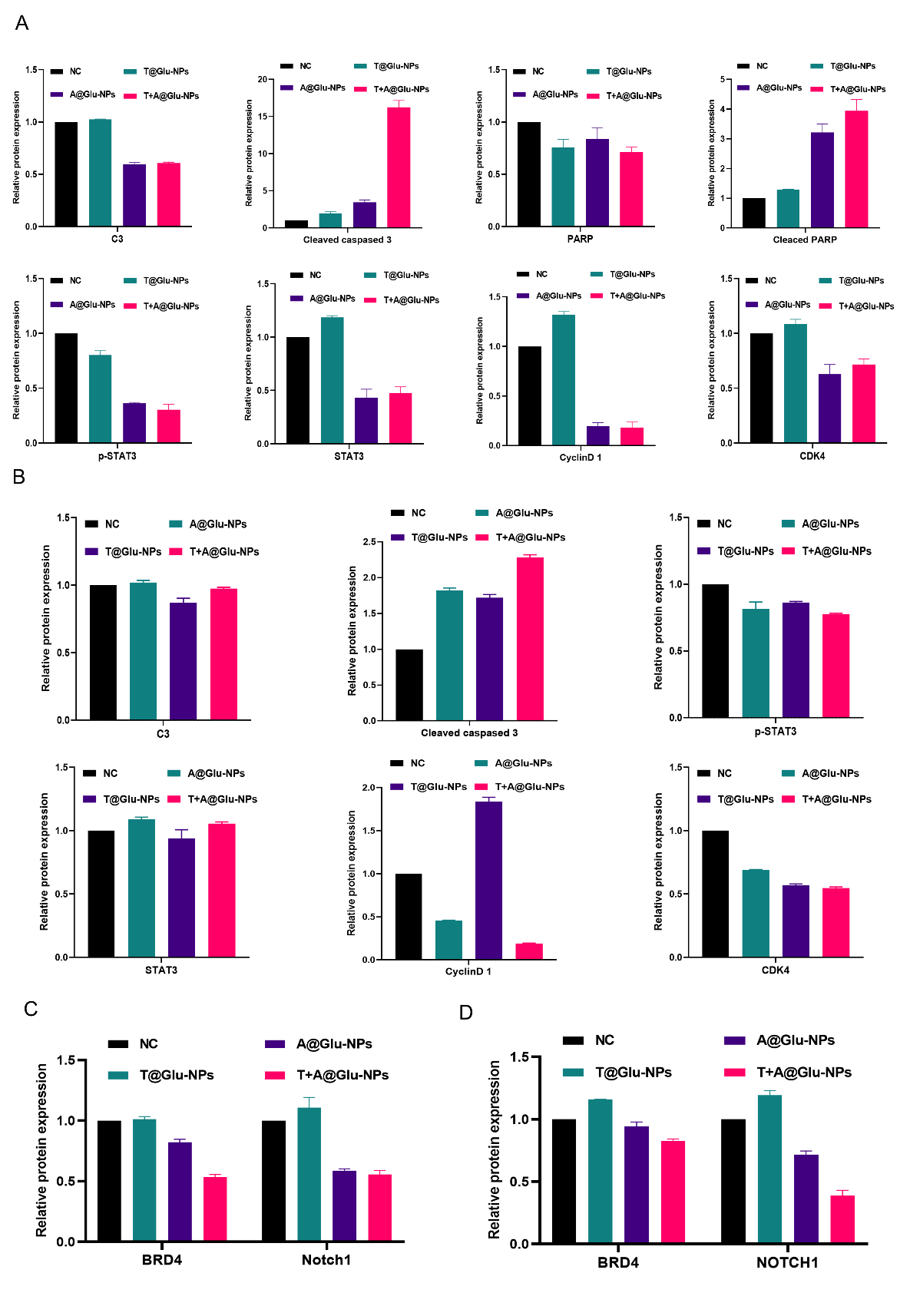


**Figure S22.** Quantitative analysis of Western Blot (A) and (C) Quantitative analysis of the Western Blot experimental results of GL261 cells (n=3). (B) and (D) Quantitative analysis of the Western Blot experimental results of LN229 cells (n=3). Data are presented as mean ± SD. No significant difference is marked with ns. *P < 0.05, **P < 0.01, ***P < 0.001 and ****P < 0.0001.


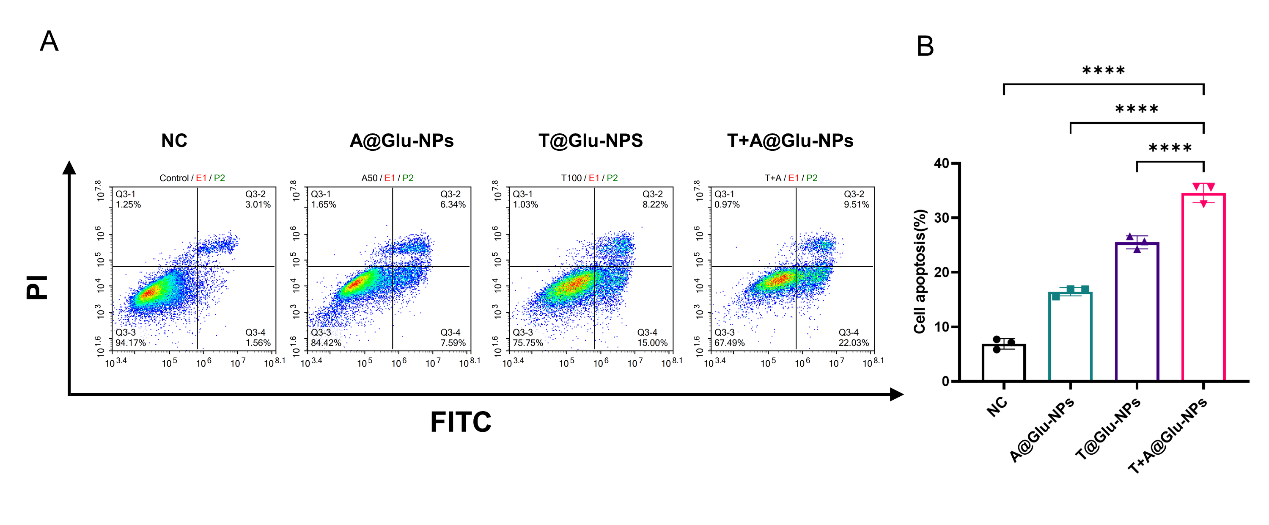


**Figure S23.** Flow apoptosis analysis of U87 cells (A) Flow apoptosis analysis of U87 cells after treated with A@Glu-NPs at 50 ng/mL, T@Glu-NPs at 100 μM and T+A@Glu-NPs for 48 h, the untreated group as a control group. (B) Statistical analysis of U87 cells (n = 3). Data are presented as mean ± SD. No significant difference is marked with ns. *P < 0.05, **P < 0.01, ***P < 0.001 and ****P < 0.0001.


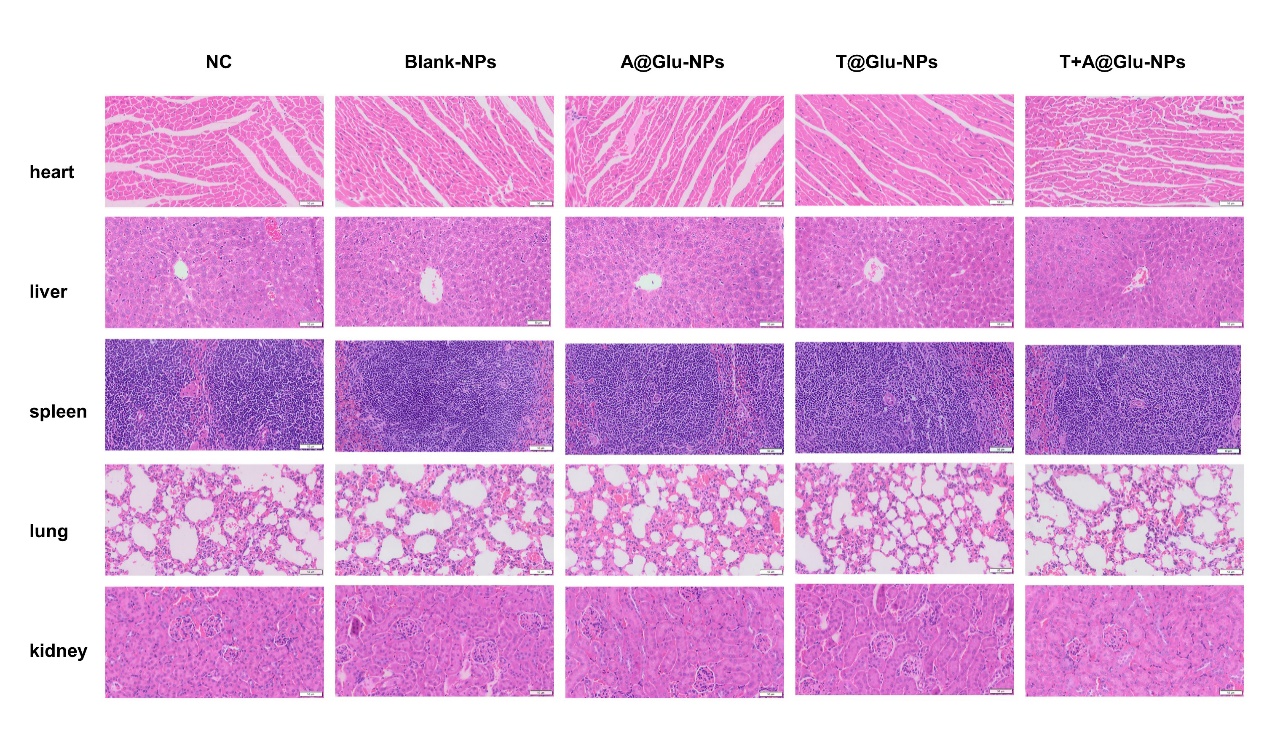


**Figure S24.** The safety assessment of GL261 orthotopic glioma mouse model. H&E staining of heart, liver, spleen, lung and kidney in GL261 orthotopic glioma mouse model (scale bar = 50 μm).


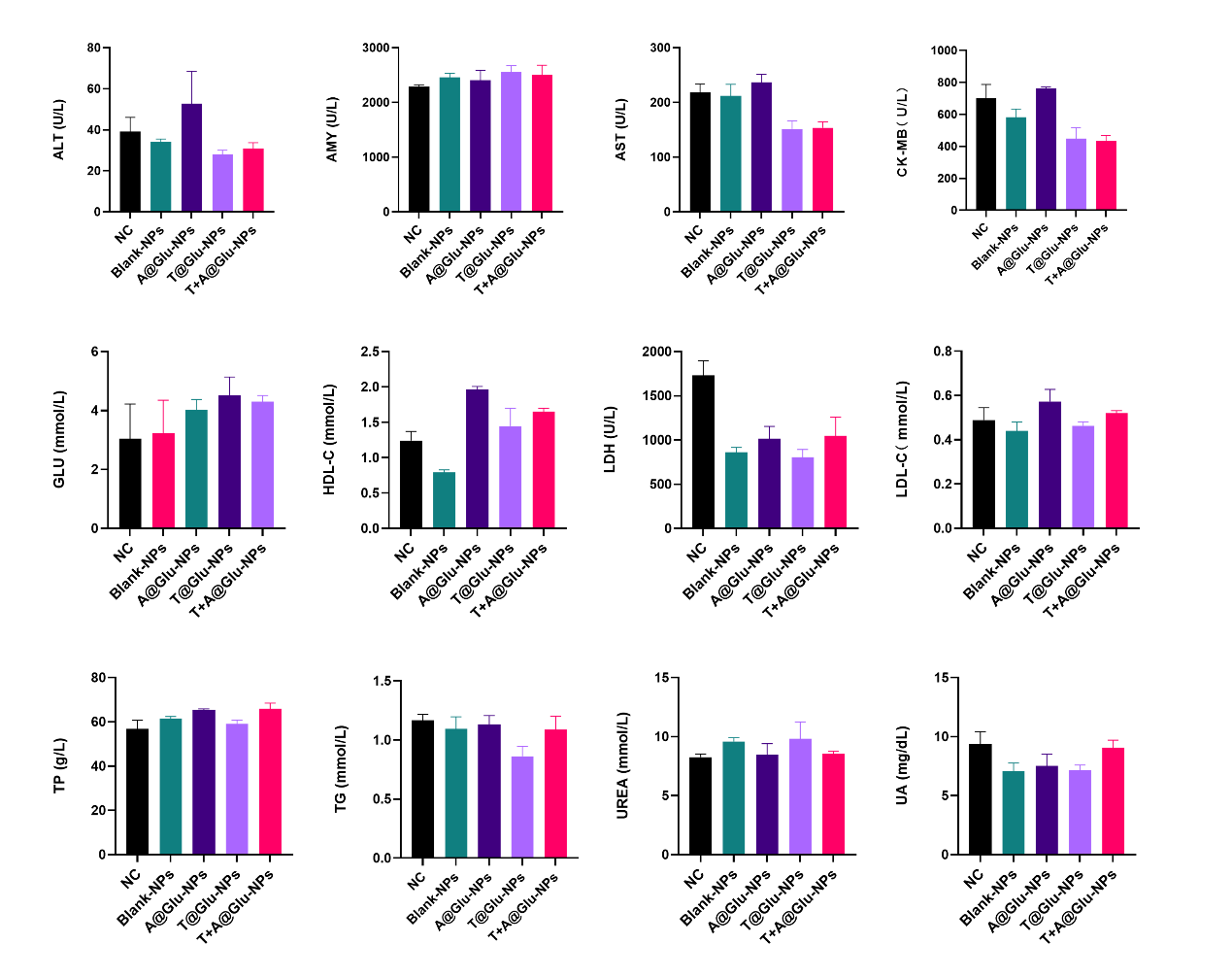


**Figure S25.** The safety assessment of GL261 orthotopic glioma mouse model. Blood biochemistry analysis of GL261 orthotopic glioma mouse model including ALT, alanine aminotransferase; AMY, amylase; AST, aspartate transaminase; CK-MB, creatine kinase isoenzymes; GLU, glucose; HDL-c, high-density lipoprotein-cholesterol; LDH, lactate dehydrogenase; LDL-c, low-density lipoprotein-cholesterol; TG, triglycerides; TP, total protein; UA, uric acid; UREA, urea. Data are presented as mean ± SD.


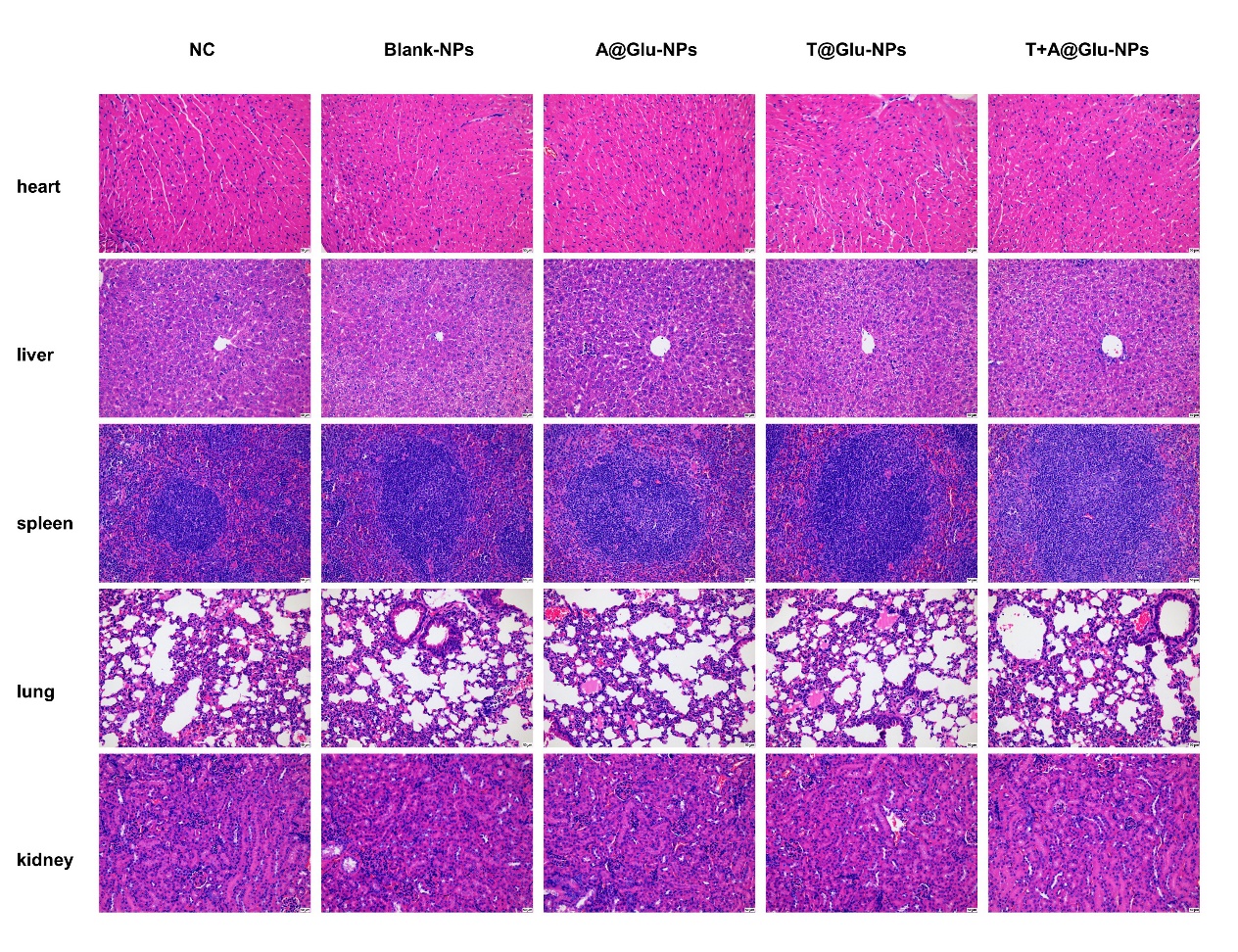


**Figure S26.** The safety assessment of GL261 subcutaneous glioma mouse model. H&E staining of heart, liver, spleen, lung and kidney in GL261 subcutaneous glioma mouse model (scale bar = 50 μm).


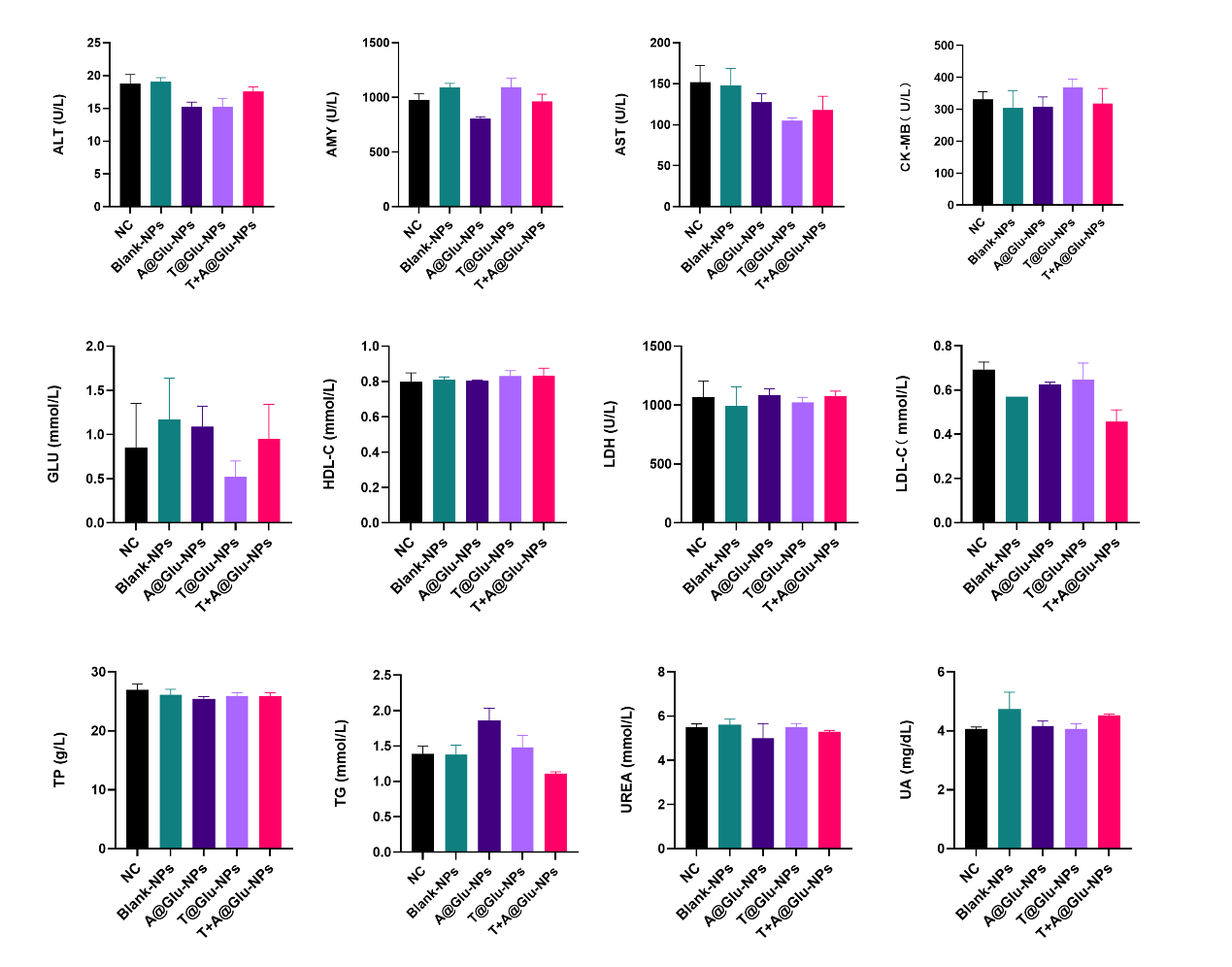


**Figure S27.** The safety assessment of GL261 subcutaneous glioma mouse model. Blood biochemistry analysis of GL261 subcutaneous glioma mouse model including ALT, alanine aminotransferase; AMY, amylase; AST, aspartate transaminase; CK-MB, creatine kinase isoenzymes; GLU, glucose; HDL-c, high-density lipoprotein-cholesterol; LDH, lactate dehydrogenase; LDL-c, low-density lipoprotein-cholesterol; TG, triglycerides; TP, total protein; UA, uric acid; UREA, urea. Data are presented as mean ± SD.


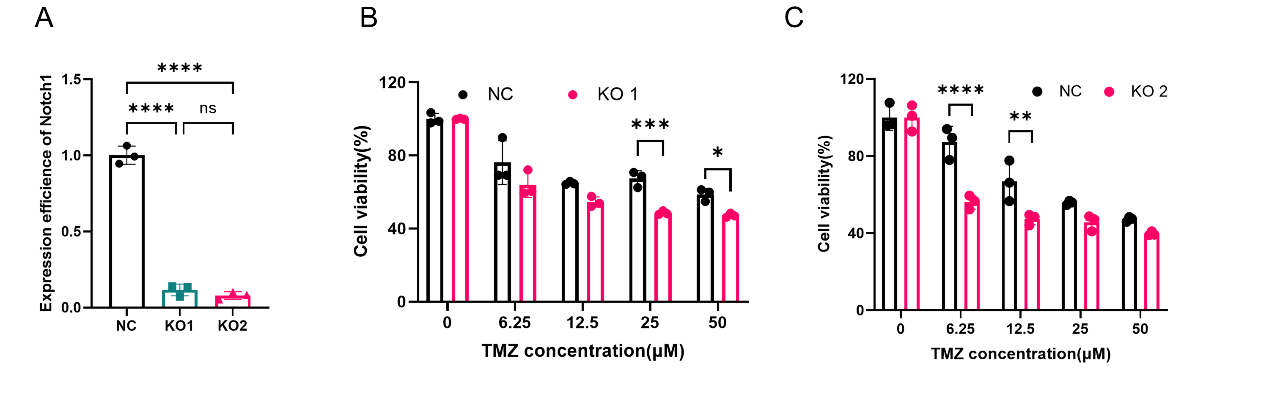


**Figure S28.** The RT-qPCR and cell viability assay of NOTCH1 gene knockout LN229 cell lines (A) The RT-qPCR of NOTCH1 gene knockout LN229 cell lines, KO1 and KO2. (B) The cell viability assay of NOTCH1 gene knockout LN229 cell line, KO1. (B) The cell viability assay of NOTCH1 gene knockout LN229 cell line, KO2 (n=3). Data are presented as mean ± SD. No significant difference is marked with ns. *P < 0.05, **P < 0.01, ***P < 0.001 and ****P < 0.0001.


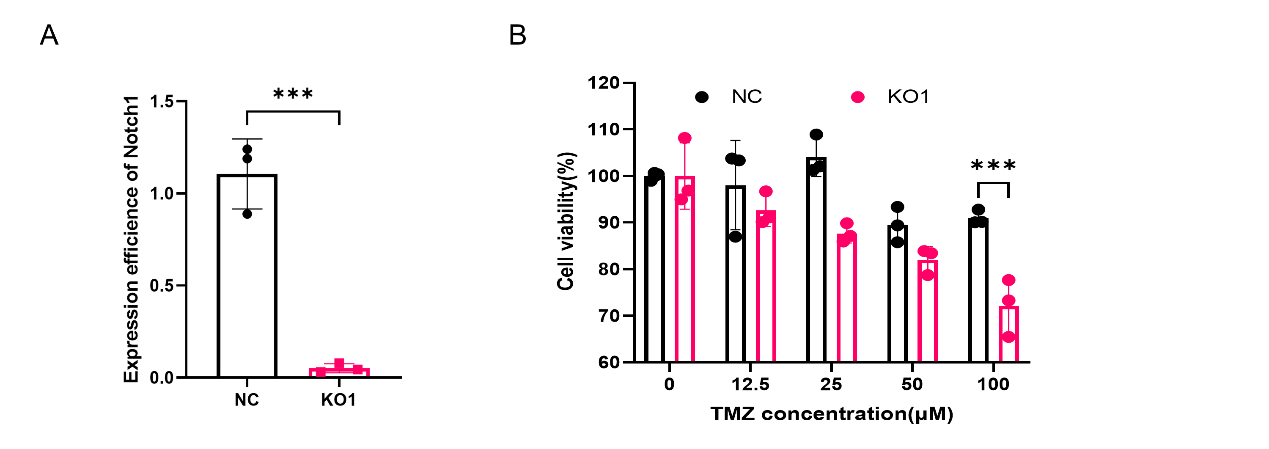


**Figure S29.** The RT-qPCR and cell viability assay of NOTCH1 gene knockout U251 cell lines (A) The RT-qPCR of NOTCH1 gene knockout U251 cell lines, KO1. (B) The cell viability assay of NOTCH1 gene knockout U251 cell line, KO1(n=3). Data are presented as mean ± SD. No significant difference is marked with ns. *P < 0.05, **P < 0.01, ***P < 0.001 and ****P < 0.0001.
